# Efficacy and Mechanisms of Alkaloids Against Enterovirus A71: A Systematic Review and Meta-Analysis of Preclinical Evidence Integrated with Network Pharmacology and Molecular Docking

**DOI:** 10.3390/ijms27156571

**Published:** 2026-07-23

**Authors:** Wenzhan Xie, Linxi Lv, Tian Wang, Jialong Wei, Yanshan Gui, Wei Gu, Hui Feng

**Affiliations:** 1 Center of Smart Laboratory and Molecular Medicine, School of Medicine, Chongqing University, Chongqing 400044, China; 202437021066t@stu.cqu.edu.cn (W.X.); 2023371008g@stu.cqu.edu.cn (L.L.); 202337021003@stu.cqu.edu.cn (J.W.); 202437131079@stu.cqu.edu.cn (Y.G.); 2 College of Chemistry and Chemical Engineering, Southwest University, Chongqing 400715, China; wangtian211002@163.com

**Keywords:** alkaloids, enterovirus A71, meta-analysis, network pharmacology

## Abstract

Enterovirus A71 (EV-A71) is a major causative agent of hand, foot, and mouth disease, yet no specific antiviral therapy has been approved. This study systematically evaluated the efficacy and mechanisms of alkaloids against EV-A71 infection by integrating meta-analysis, network pharmacology, and molecular docking. Nine animal studies were included. Meta-analysis suggested that alkaloid intervention significantly improved survival (OR = 30.62, 95% CI: 10.44–89.82), reduced clinical severity, attenuated body weight loss, and decreased viral loads in infected tissues. Subgroup analyses preliminarily suggested that quinolizidine alkaloids and high-dose regimens (>5 mg/kg) may be associated with preclinical intervention effects. Network pharmacology predicted 155 shared targets between seven active alkaloids and EV-A71-related genes, with MAPK1, MAPK3, JUN, AURKB, and MAPK8 recognized as core targets through computational screening. Functional enrichment analysis suggested significant involvement of the MAPK, TNF, and IL-17 signaling pathways. Molecular docking provided computational support for stable binding affinities between active alkaloids and core targets (−6.7 to −9.3 kcal/mol). Collectively, these findings suggest that alkaloids exert anti-EV-A71 effects through both direct antiviral activity and host-directed regulatory mechanisms, supporting their potential as candidates for the development of novel anti-EV-A71 therapeutics.

## 1. Introduction

Enterovirus A71 (EV-A71) is a non-enveloped, positive-sense single-stranded RNA virus of the Picornaviridae family, with a 7.4 kb genome consisting of a 5′ untranslated region (UTR), a single open reading frame, and a 3′ UTR [1]. Its genome undergoes internal ribosomal entry site (IRES)-dependent translation into a polyprotein, which is proteolytically cleaved into 4 structural proteins (VP1-VP4) and 7 non-structural proteins (2A, 2B, 2C, 3A, 3B, 3C, 3Dpol) [2]. Recent reviews have comprehensively summarized the structure, replication cycle, and host–pathogen interactions of EV-A71, providing important insights into antiviral target discovery [3,4]. EV-A71 is the primary causative pathogen of hand, foot, and mouth disease (HFMD), a typically mild, self-limiting childhood infection presenting with fever, rash, and oral ulcers [5,6,7]. However, a small proportion (1.1%) of patients rapidly develop neurological and systemic complications that may be fatal, such as aseptic meningitis (AM), brainstem encephalitis (BSE), acute flaccid paralysis (AFP), neurogenic pulmonary edema (NPE), cardiopulmonary failure, and even death [8,9,10,11]. Neurological injury-induced pulmonary edema and hemorrhage are the leading causes of EV-A71-related death, with over 70% of severe and 93% of fatal HFMD cases linked to EV-A71 infection [12]. Since its first isolation in 1969, EV-A71 has caused recurrent large outbreaks worldwide, especially in the Asia-Pacific region, posing a persistent global public health threat [9,12,13,14,15].

Despite this substantial disease burden, no specific anti-EV-A71 therapies are approved globally. Three inactivated monovalent EV-A71 vaccines approved in China only protect against the homologous strain [16], with no cross-protection against other enteroviruses or therapeutic effect on established infection [17]. The broad-spectrum antiviral ribavirin has shown unsatisfactory efficacy against EV-A71 with notable pediatric adverse effects [18,19], and is not approved for this indication [20]. Furthermore, treatment for EV-A71 infection is limited to supportive care, as direct antiviral therapies have shown little efficacy in severe cases of EV-A71 infection. Thus, there is an urgent unmet need for safe and effective anti-EV-A71 therapeutics.

Plant-derived natural products have long served as a rich and valuable source for antiviral drug discovery [21], among which alkaloids represent one of the promising classes of natural products [22]. Alkaloids are a large and diverse group of naturally occurring organic compounds, with approximately 20,000 known compounds identified to date, the majority of which are isolated from higher plants [23,24]. The characteristic nitrogen-containing heterocyclic structure of alkaloids confers their inherent alkalinity and diverse pharmacological properties [25]. Alkaloids exhibit a wide spectrum of biological activities, including antiviral, anti-tumor, anti-depressant, anti-inflammatory, anti-angiogenic, and neuroprotective effects, and have been successfully developed into multiple clinical therapeutic agents [26,27,28] owing to their favorable physicochemical properties (water solubility under acidic conditions and lipophilicity under neutral/alkaline conditions). In recent years, accumulating preclinical studies have demonstrated that a variety of natural alkaloids, including berberine, cepharanthine, lycorine and its derivatives, exert significant anti-EV-A71 activity in both in vitro cell models and in vivo animal studies [29]. Their underlying mechanisms of action mainly include targeting viral entry [30], inhibiting viral adsorption and replication [31], blocking viral protein synthesis [32], regulating host oxidative stress and immune responses [33,34], and alleviating virus-induced tissue damage [32,34]. However, most existing studies are single-center preclinical trials with small sample sizes, and the reported efficacy varies substantially across studies. More importantly, no systematic review and meta-analysis have been conducted to quantitatively synthesize the preclinical efficacy evidence of natural alkaloids against EV-A71 infection, and their multi-target mechanisms of action remain largely unelucidated.

To address these critical research gaps, the present study integrated network pharmacology analysis and meta-analysis to comprehensively evaluate the efficacy of natural alkaloids against EV-A71 infection and elucidate their underlying multi-target mechanisms of action. Our findings provide robust preclinical evidence and systematic theoretical basis for the development and clinical translation of alkaloid-based anti-EV-A71 therapeutic strategies.

## 2. Methods

### 2.1. Meta Analysis

This systematic review and meta-analysis were conducted and reported in accordance with the PRISMA 2020 statement (Supplementary Materials) [35]. The protocol, utilizing SYRCLE’s tool for animal studies [36], is registered in PROSPERO under registration number CRD420261336466.

#### 2.1.1. Search Strategy

A comprehensive literature search was conducted in the following electronic databases from inception to March 2026: MEDLINE via PubMed (U.S. National Library of Medicine), Web of Science Core Collection (Clarivate Analytics), Embase (Elsevier) [37], the Cochrane Library (Wiley) [38], Scopus (Elsevier), China National Knowledge Infrastructure (CNKI), VIP Database (CQVIP), and Wanfang Data. The full characteristics of all included databases are summarized in Table S1.

Medical Subject Headings (MeSH) terms and free-text keywords related to “Human Enterovirus A” and “Alkaloids” were used in the literature search, with targeted customization of the search strategy for each database (Table S2). No restrictions on language or publication year were applied. Supplementary references, including unpublished studies, were manually screened to identify eligible literature.

#### 2.1.2. Inclusion and Exclusion Criteria

Studies were included based on the PICOS framework, P (population): in vivo animal models with Enterovirus 71 (EV-A71) infection; I (intervention): alkaloid compound intervention; C (control): vehicle, normal saline, phosphate-buffered saline (PBS), or no intervention; O (outcomes) primary outcome: survival rate, secondary outcomes: clinical score, body weight, and viral replication; S (study design): controlled animal studies.

Exclusion criteria were as follows: (1) studies that clearly did not meet the predefined inclusion criteria upon initial screening; (2) incomplete or unavailable data for meta-analysis; (3) secondary literature types (reviews, abstracts, etc.); and (4) studies without any of the prespecified relevant outcome data.

#### 2.1.3. Data Extraction and Quality Assessment

Data extraction was performed independently by two reviewers using a pre-designed standardized spreadsheet. The extracted information included first author/publication year, characteristics of experimental animals (species, strain, age), details of EV-A71 infection animal model construction (virus strain type, inoculation dose, inoculation route), details of intervention measures (alkaloid type and specific name, administration dose, administration frequency, intervention duration, administration route), control group intervention protocol, and outcome measures. For studies investigating multiple compounds, data were extracted from the intervention arm corresponding to the primary focus of the original article. If results were presented only in graphical form, we contacted the corresponding authors of the relevant studies to obtain raw data. If this was not possible, WebPlotDigitizer (v4.7) was used to quantify the results. Furthermore, when extracting tissue viral load data, all units were converted to a log10 scale. For viral load measurements below the limit of detection (LOD), an imputation method using LOD/2 was employed [39]. Discrepancies in data extraction were resolved through discussion or, when necessary, by consultation with a third reviewer.

Two assessors independently performed quality assessments on the included studies by applying the SYRCLE RoB (Risk of Bias) tool (https://www.radboudumc.nl/en/research/departments/health-evidence/syrcle, accessed on 15 March 2026), which is specifically designed for animal intervention research [36]. Entries in this tool are as follows: selection bias (sequence generation, baseline characteristics, and allocation concealment), performance bias (random accommodation and blinding), detection bias (random outcome assessment and blinding), attrition bias (incomplete outcome data), reporting bias (selective outcome reporting), and other sources of bias. Each domain was rated as low risk (+), unclear risk (?), or high risk (−). The two researchers conducted the bias evaluation independently. Any discrepancies in scoring were resolved through discussion with a third researcher. Additionally, the Grading of Recommendations, Assessment, Development, and Evaluation (GRADE) approach (https://gdt.gradepro.org/app/, accessed on 25 March 2026) was employed to assess methodological quality [40,41].

#### 2.1.4. Statistical Analysis

Data analysis and visualization were performed using Review Manager 5.4 (Cochrane Collaboration, Oxford, UK). Effect measures were applied according to the data type. For dichotomous outcomes, odds ratios (ORs) with 95% confidence intervals (CIs) were calculated and pooled using the Mantel–Haenszel method. For continuous variables, mean differences (MD) with 95% CI were calculated. A *p*-value < 0.05 was considered statistically significant. Heterogeneity among studies was assessed using the Chi-square test (χ^2^) and the I^2^ statistic. A fixed-effect model was applied when *p* > 0.1 and I^2^ < 50%, indicating low heterogeneity [42,43,44]; otherwise, a random-effects model was used [45]. Pre-specified subgroup analyses were performed to explore potential sources of heterogeneity and evaluate the impact of intervention characteristics on intervention efficacy. Subgroup analyses were conducted based on alkaloid type (isoquinoline vs. quinolizidine alkaloids) and dose (≤5 mg/kg vs. >5 mg/kg), provided that each subgroup contained at least one study. Sensitivity analyses were conducted for outcomes comprising more than two studies using Stata 17.0 software (StataCorp LLC, College Station, TX, USA) to investigate the influence of individual studies on the overall pooled effect size. Funnel plots and Egger’s test were pre-specified for meta-analyses including ten or more studies. However, as all analyses in this review contained fewer than ten studies, these methods were not performed. Instead, Orwin’s fail-safe N method was applied to estimate the number of unpublished null-effect studies required to reduce the pooled effect size to a trivial threshold. Since an odds ratio of 1.0 indicates no effect, we conservatively selected an OR of 1.1 as the trivial effect size for the present analysis. A larger fail-safe N value indicates greater robustness of the findings to potential publication bias [46].

### 2.2. Network Pharmacology

#### 2.2.1. Compound Target Prediction

Seven compounds, namely berberine, cepharanthine, emetine, harmine, lycorine, matrine, and oxysophocarpine, were screened through systematic meta-analysis. Notably, lycorine derivative 7e and lycorine derivative LY-55 were excluded from network pharmacology prediction because standardized structural identifiers and canonical simplified molecular input line entry system (SMILES) for lycorine derivatives 7e and LY-55 were unavailable in the public databases used in this study; native lycorine was included only as a surrogate compound for exploratory network pharmacology analysis. This substitution should not be interpreted as evidence that these derivatives share identical molecular targets or pharmacological mechanisms with lycorine. The canonical SMILES of each compound was obtained from the PubChem database. Potential human targets of these compounds were predicted using the SwissTargetPrediction web server (http://www.swisstargetprediction.ch/, accessed on 5 April 2026) with Homo sapiens selected as the species. All predicted targets were combined, and duplicate entries were removed to generate a non-redundant target list for subsequent analysis (Table S3).

#### 2.2.2. Identification of Disease-Related Targets

To comprehensively obtain disease targets associated with EV-A71 infection, the DisGeNET database (https://www.disgenet.org/, accessed on 5 April 2026) and GeneCards database (https://www.genecards.org/, accessed on 5 April 2026) were searched in March 2026. In DisGeNET, the keyword “Enterovirus A71” was used with a screening criterion score ≥ 0.2. In GeneCards, the keywords “Enterovirus A71”, “Enterovirus infection”, and “Hand, foot and mouth disease” were used with a screening criterion relevance score ≥ 5. The search results from different databases and keywords were merged, and duplicate entries were removed to generate a non-redundant list of EV-A71-related disease targets, yielding a total of 1768 genes. A rigorous, stepwise protocol was applied to screen and integrate targets from these heterogeneous sources (Table S4).

#### 2.2.3. Identification of Intersection Targets

The compound-related targets and disease-related targets were intersected to identify potential therapeutic targets of the seven compounds against EV-A71 infection. The overlapping targets were visualized using a Venn diagram generated with the VennDiagram package in R software (version 4.3.2). A total of 155 common targets were identified and subjected to subsequent PPI network construction and functional enrichment analyses (Table S5).

#### 2.2.4. Construction of Protein-Protein Interaction (PPI) Network

The common drug-disease targets were imported into the STRING database (https://string-db.org/, accessed on 5 April 2026). The organism was set to Homo sapiens, and the minimum required interaction score was set to 0.4 to construct the PPI network, with disconnected nodes hidden. The interaction data were downloaded in tab-separated values (TSV) format, and the network was visualized using Cytoscape software (version 3.10.4). The CytoHubba plugin was applied to calculate four centrality measures, namely Maximal Clique Centrality (MCC), Betweenness Centrality (BC), Closeness Centrality (CC_net), and Degree Centrality (DC), to identify hub genes in the network (Table S6).

#### 2.2.5. Screening of Core Targets and Network Analysis

Core targets were screened based on the compound–target interaction network. First, the CytoHubba plugin (The Cytoscape Consortium, San Diego, CA, USA) was used to calculate four centrality measures (MCC, BC, CC_net, DC) in the PPI network. However, the hub genes identified by PPI topology alone were not compound-targeted genes. Therefore, a pharmacological relevance filter was applied: genes that were targeted by at least two compounds and played central roles in the network were identified as core pharmacological targets, yielding five genes: MAPK1, MAPK3, JUN, AURKB, and MAPK8. Of note, the degree values in this context represent the number of active compounds targeting each gene (compound–target network degree), not the number of protein–protein interactions in the PPI network.

The yFiles Layouts plugin in Cytoscape (The Cytoscape Consortium, San Diego, CA, USA) was used for hierarchical layout. Node size was proportional to the degree value, and node colors were distinguished by node type (compound, target, disease, pathway). Finally, the compound–target–pathway network was constructed and visualized.

#### 2.2.6. Functional Enrichment Analysis

To elucidate the biological functions of the 155 common targets, Gene Ontology (GO) functional enrichment analysis was performed using the clusterProfiler package (version 4.8.0) in R software (version 4.3.2). The analysis covered three categories: biological process (BP), cellular component (CC_GO), and molecular function (MF). The species was set to Homo sapiens, and the significance threshold was set at *p* < 0.05, adjusted by the Benjamini–Hochberg (BH) method for multiple testing correction. The top 10 enriched terms from each category were visualized using a bar plot, with BP, CC_GO, and MF distinguished by green, blue, and red colors, respectively. Kyoto Encyclopedia of Genes and Genomes (KEGG) pathway enrichment analysis was performed using the clusterProfiler package (version 4.10.0). The top 20 enriched pathways were visualized as a bubble plot, and the complete list of significantly enriched pathways (*p*.adjust < 0.05) is provided in Table S7.

For network visualization, pathways were prioritized based on three criteria: (1) statistical significance (adjusted *p*-value < 0.05), (2) biological relevance to EV-A71 infection (viral infection, immune response, inflammation, or apoptosis), and (3) connectivity to at least one of the five core targets (MAPK1, MAPK3, JUN, AURKB, MAPK8). The selection rationale for the 10 visualized pathways is detailed in Supplementary Table S8.

### 2.3. Molecular Docking Verification

The three-dimensional structures of core target proteins (MAPK1, MAPK3, JUN, AURKB, MAPK8) were obtained from the RCSB protein data bank (PDB) database. The 3D structures of active compounds were downloaded from the PubChem database. Water molecules and original ligands were removed from protein structures, and hydrogen atoms and charges were added using PyMOL 2.5 software. The docking pocket was defined according to the position of the original ligand or the active center of the target protein. Molecular docking was performed using the CB-Dock web server. The binding affinity was expressed in kcal/mol, and values ≤ −6.0 kcal/mol were defined as stable binding. The 3D binding conformations and 2D interaction diagrams were visualized and analyzed using PyMOL 2.5 and Maestro 12.8 software. Furthermore, oxysophocarpine was excluded from docking as it only targeted one core target (MAPK1) with a predicted affinity (−5.8 kcal/mol) slightly above the −6.0 kcal/mol threshold.

## 3. Results

### 3.1. Meta Analysis

**Figure 1:**
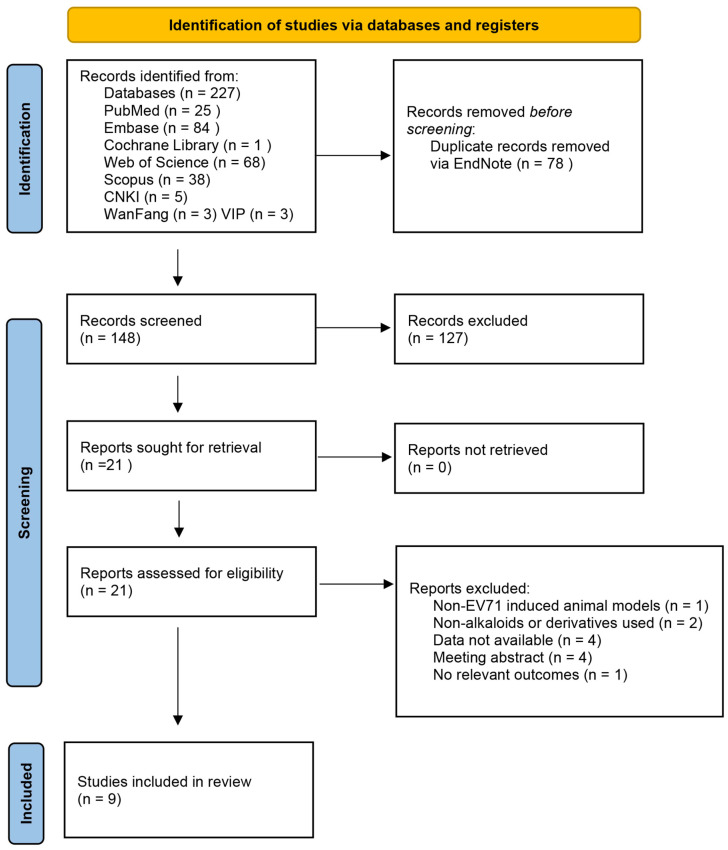
PRISMA flowchart and study selection.

A total of 227 studies were identified through the database search. After removing duplicates, 148 studies were retained for further analysis. Following title and abstract screening, 21 full-text articles were assessed for eligibility. Ultimately, nine studies met the inclusion criteria and were included in this systematic review and meta-analysis. The study selection process, along with the reasons for exclusion, is illustrated in Figure 1.

#### 3.1.1. Study Selection and Characteristics

The basic characteristics of the nine studies ultimately included in this systematic review are summarized in Table 1. All studies were published between 2011 and 2025. All included studies established an EV-A71 infection animal model using mice. The mouse strains employed encompassed ICR mice, KM mice, AG129 mice, and BALB/c mice, with ages ranging from neonatal mice within 24 h of birth, 7–14-day-old infant mice, to adult mice aged 5 weeks to 3 months. In all studies, EV-A71 infection was induced via intraperitoneal (i.p.) injection. The viral strains used included different subtypes such as EV-A71 MP4, C4, MP10, GZ-CII, H-MA, and Gluc-EV-A71, with inoculation titers ranging from 1 × 10^6^ 50% tissue culture infectious dose (TCID_50_) to 1.2 × 10^8^ plaque forming unit (PFU). The interventions in the included studies were all natural alkaloids (Table 2), which can be categorized into three main classes: isoquinoline alkaloids (cepharanthine, berberine, emetine, lycorine derivative 7e, lycorine derivative ly-55, lycorine), indole alkaloids (harmine), and quinolizidine alkaloids (oxymatrine, matrine). The dosage of drug administration ranged from 0.20 mg/kg to 20 mg/kg. The primary route of administration was intraperitoneal injection, with only one study employing intragastric (i.g.) administration [47]. Intervention regimens included pre-intervention before EV-A71 infection, post-infection intervention, and a combination of pre-intervention and continuous post-infection administration. The duration of intervention ranged from 4 to 14 days. All studies reported outcomes related to EV-A71 infection, with survival rate, clinical symptom score, and viral load being the most commonly assessed indicators.

#### 3.1.2. Risk of Bias Assessment

**Figure 2:**
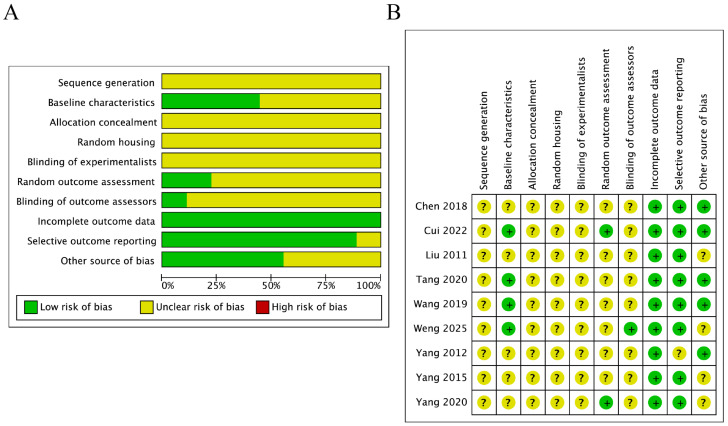
The risk of bias assessment of the 9 studies included in this meta-analysis based on the SYRCLE’s Risk of Bias tool. “+”, low risk of bias; “?”, unclear risk of bias. (**A**) Risk of bias graph; (**B**) Risk of bias summary [30,32,33,34,47,48,49,50,51].

The methodological quality of the nine included studies was assessed using the SYRCLE Risk of Bias tool (https://www.radboudumc.nl/en/research/departments/health-evidence/syrcle, accessed on 15 March 2026), with detailed results presented in Figure 2 and summarized in Table S9. The reporting of key methodological details was generally incomplete across the included studies. None of the studies adequately described the specific method used for random sequence generation, and only four studies explicitly reported the comparability of baseline characteristics between groups. Allocation concealment, random housing, and blinding of personnel were not reported in any of the studies. Regarding detection bias, only two studies reported random outcome assessment, and merely one study implemented blinding of outcome assessors. All studies completely reported outcome data, and only one study exhibited deficiencies related to selective outcome reporting. No study was judged as having a high risk of bias in any domain. However, inadequate reporting was prevalent in core methodological domains such as randomization, allocation concealment, and blinding, indicating significant methodological limitations in the available evidence. These deficiencies reduced the overall certainty of the evidence. As assessed by the GRADE approach, the certainty of evidence for the primary outcome was moderate, while the certainty of evidence for secondary outcomes was rated as low to very low due to serious risk of bias, substantial inconsistency, and indirectness (Table S10). These ratings reflect the exploratory and hypothesis-generating nature of the secondary findings.

#### 3.1.3. Quantitative Synthesis (Meta-Analysis)

It is important to note that these pooled analyses combine data from different alkaloid classes (isoquinoline, indole, and quinolizidine alkaloids) with distinct pharmacological properties. The large OR should be interpreted with caution, as it represents an aggregate measure across diverse compounds and should not be construed as evidence of a uniform class effect.

##### Survival Rate

**Figure 3:**
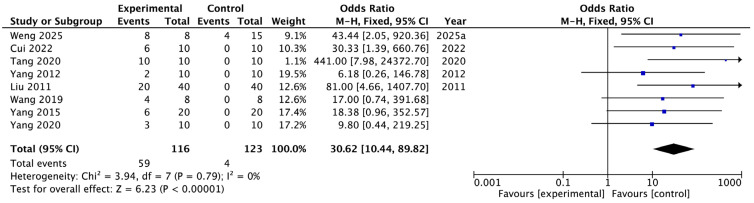
Forest plot of the effect of alkaloid intervention on survival rate in EV-A71-infected animal models [30,32,33,34,47,48,50,51].

The analysis of eight studies [30,32,33,34,47,48,50,51] provides evidence that alkaloid intervention improved survival in EV-A71-infected animals compared with the controls [OR: 30.62 (95% CI: 10.44, 89.82), *p* < 0.00001; heterogeneity: I^2^ = 0%, *p* = 0.79, GRADE of evidence: moderate; Figure 3]. While this OR suggests a strong statistical association in the pooled analysis, the magnitude of the effect should be interpreted with caution. This large OR is likely inflated by small sample sizes and zero-event studies in the included mouse models, and it should not be interpreted as a direct quantitative prediction of clinical efficacy in humans.

##### Clinical Symptoms

**Figure 4:**
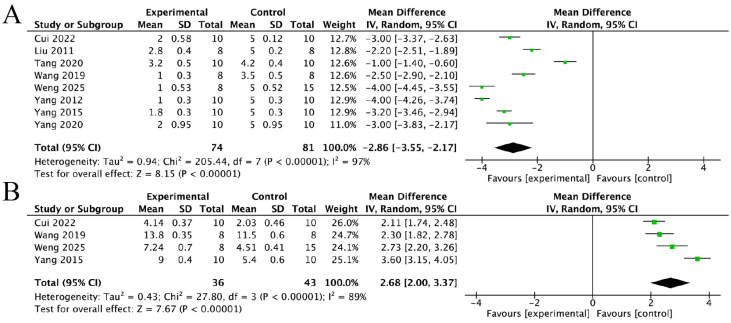
Forest plots of the effect of alkaloid intervention on clinical outcomes in EV-A71-infected animal models. (**A**) Clinical score [30,32,33,34,47,48,50,51]. (**B**) Body weight [30,33,34,50].

The analysis of eight studies [30,32,33,34,47,48,50,51] provides evidence that alkaloid intervention reduced clinical severity in EV-A71-infected animals compared with the controls [MD: −2.86 (95% CI: −3.55, −2.17), *p* < 0.00001; heterogeneity: I^2^ = 97%, *p* < 0.00001, GRADE of evidence: low; Figure 4A]. Data from four studies [30,33,34,50] indicated an attenuation of EV-A71-induced body weight loss associated with alkaloid intervention [MD: 2.68 (95% CI: 2.00, 3.37), *p* < 0.00001; heterogeneity: I^2^ = 89%, *p* < 0.00001, GRADE of evidence: very low; Figure 4B].

##### Viral Replication

**Figure 5:**
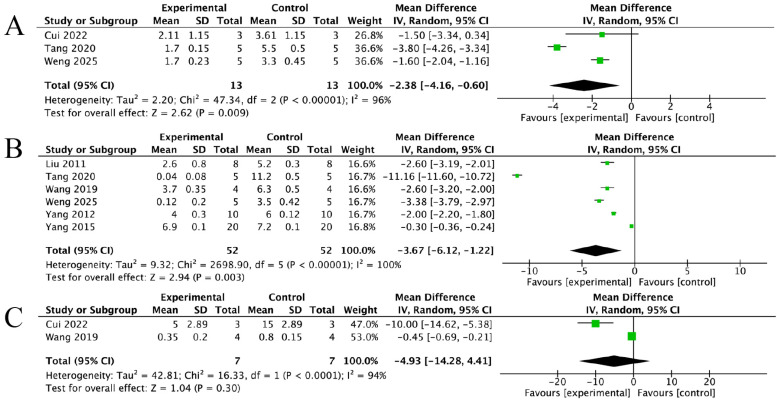
Forest plots of the effect of alkaloid intervention on viral replication in EV-A71-infected animal models. (**A**) Viral load in brain tissue [30,33,47]. (**B**) Viral load in hind limb muscle tissue [30,32,34,47,50,51]. (**C**) VP1 protein expression [33,34].

The analysis of three studies [30,33,47] provides evidence that alkaloid intervention reduced viral load in the brain tissue of EV-A71-infected animals compared with the controls [MD: −2.38 (95% CI: −4.16, −0.60), *p* = 0.009; heterogeneity: I^2^ = 96%, *p* < 0.00001, GRADE of evidence: very low; Figure 5A]. Data from six studies [30,32,34,47,50,51] indicated a decrease in viral load in hind limb muscle associated with alkaloid intervention [MD: −3.67 (95% CI: −6.12, −1.22), *p* = 0.003; heterogeneity: I^2^ = 100%, *p* < 0.00001, GRADE of evidence: low; Figure 5B]. Furthermore, analysis of two studies [33,34] showed no significant effect of alkaloid intervention on VP1 protein expression [MD: −4.93 (95% CI: −14.28, 4.41), *p* = 0.30; heterogeneity: I^2^ = 94%, *p* < 0.00001, GRADE of evidence: very low; Figure 5C].

##### Subgroup Analysis

**Figure 6:**
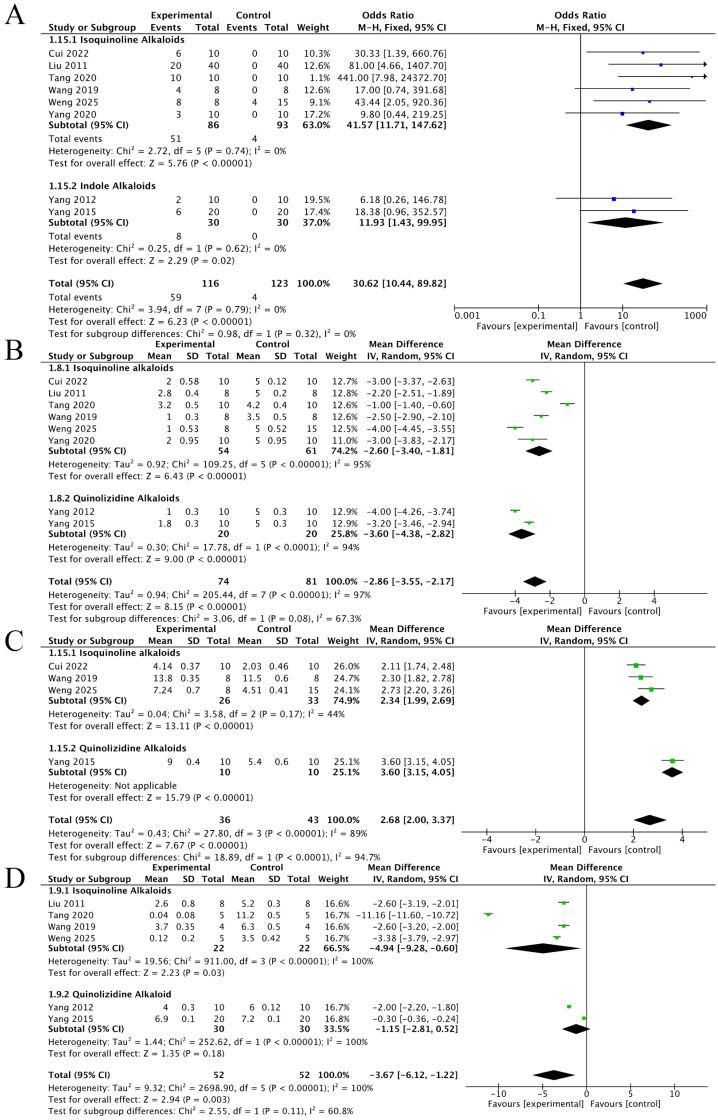
Subgroup analyses of the effect of alkaloid intervention on outcomes based on alkaloid type (isoquinoline alkaloids vs. quinolizidine alkaloids). (**A**) Survival rate [30,32,33,34,47,48,50,51]. (**B**) Clinical severity score [30,32,33,34,47,48,50,51]. (**C**) Body weight [30,33,34,50]. (**D**) Viral load in hind limb muscle tissue [30,32,34,47,50,51].

**Figure 7:**
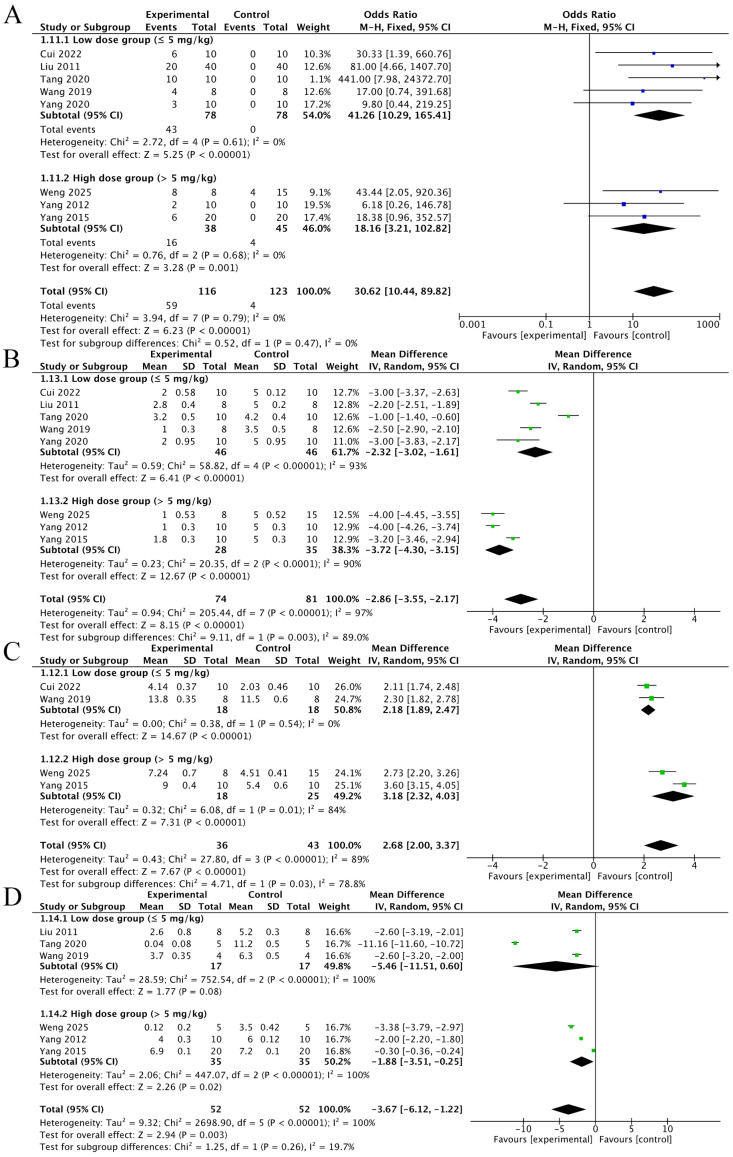
Subgroup analyses of the effect of alkaloid intervention on outcomes based on dose (low dose: ≤5 mg/kg vs. high dose: >5 mg/kg). (**A**) Survival rate [30,32,33,34,47,48,50,51]. (**B**) Clinical severity score [30,32,33,34,47,48,50,51]. (**C**) Body weight [30,33,34,50]. (**D**) Viral load in hind limb muscle tissue [30,32,34,47,50,51].

Owing to the high heterogeneity across studies and to explore the optimal intervention strategy, we performed subgroup analyses based on alkaloid type (isoquinoline alkaloids vs. quinolizidine alkaloids, Figure 6A–D) and dose (low dose: ≤5 mg/kg vs. high dose: >5 mg/kg, Figure 7A–D). Subgroup analyses revealed that alkaloid type and dose may partially explain the observed heterogeneity in clinical score and body weight, while also demonstrating that quinolizidine alkaloids and high-dose regimens showed a trend toward greater efficacy in attenuating body weight loss and reducing clinical severity compared with isoquinoline alkaloids and low-dose regimens. Notably, significant heterogeneity remained in all secondary outcomes after subgrouping, indicating that other unmeasured factors (e.g., animal strain, viral subtype) may contribute to the variability.

##### Publication Bias and Sensitivity Analysis

**Figure 8:**
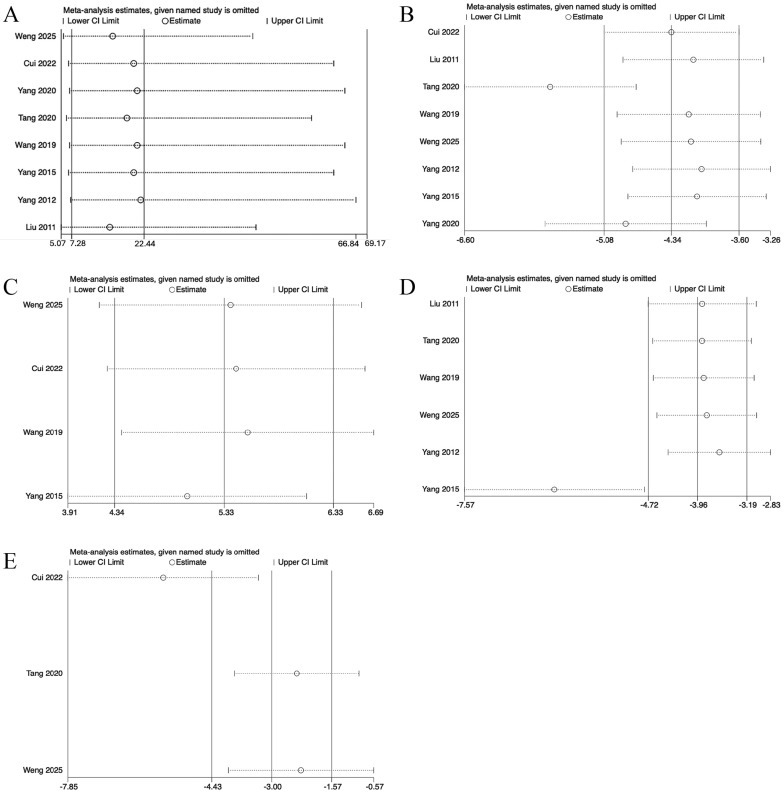
Sensitivity analysis chart. (**A**) Survival rate [30,32,33,34,47,48,50,51]. (**B**) Clinical score [30,32,33,34,47,48,50,51]. (**C**) Body weight [30,33,34,50]. (**D**) Viral load in brain tissue [30,33,47]. (**E**) Viral load in hind limb muscle tissue [30,32,34,47,50,51].

Sensitivity analysis was performed to assess the stability of pooled results by sequentially omitting each included study. For all outcomes, the pooled effect sizes and their 95% confidence intervals (CIs) remained statistically significant without crossing the null effect line, confirming the robustness of the overall conclusions (Figure 8A–E).

Owing to the fact that this meta-analysis included only 9 studies, which is below the recommended minimum of 10 studies required for funnel plot visualization and associated tests (e.g., Egger’s test), the statistical power of these tests was too low to yield reliable results. Therefore, we used Orwin’s fail-safe N method to evaluate the potential impact of publication bias on the pooled results. Setting the critical effect size (dc) to 1.1, we calculated a fail-safe N (Nfs) of 279 (Table 3). This indicates that 279 unretrieved or unpublished studies with an odds ratio of 1.1 would be required to reduce the pooled effect size (OR = 30.62) to a trivial level of 1.1. This relatively high fail-safe N suggests that the main conclusions of this study are, to some extent, robust against potential publication bias. Despite the reassuring fail-safe N, publication bias cannot be completely ruled out given the small number of included studies.

### 3.2. Network Pharmacology Analysis of Active Compounds Against EV-A71 Infection

**Figure 9:**
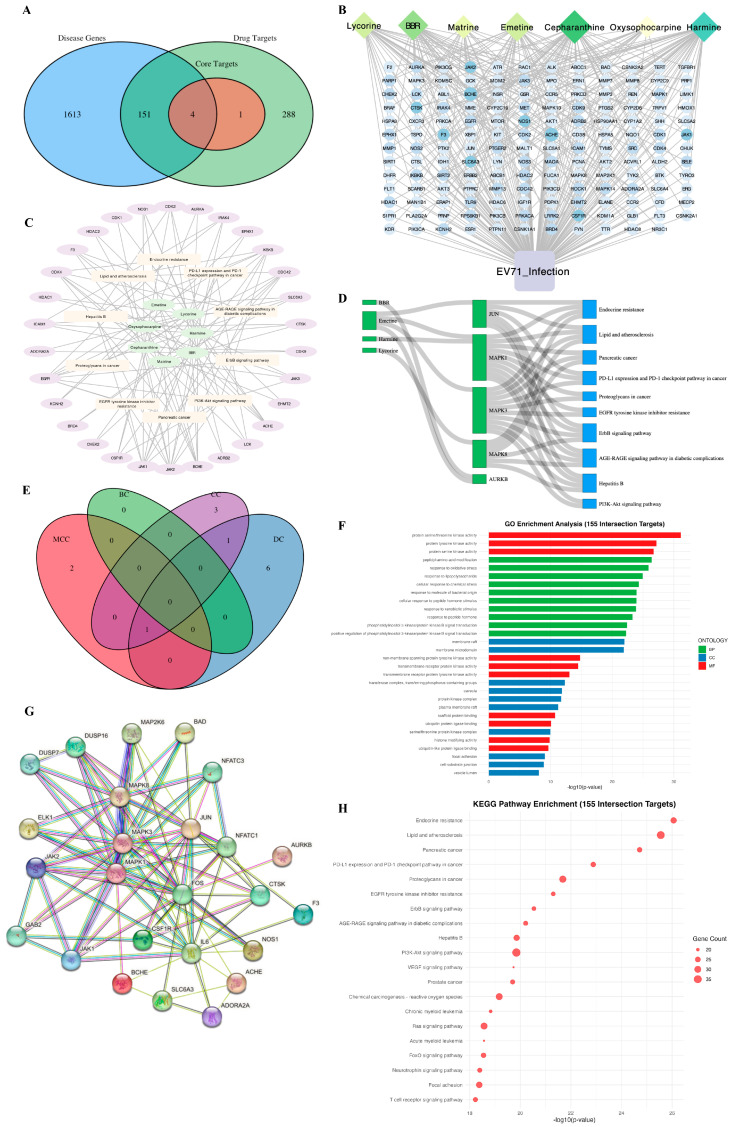
Network pharmacology analysis of the 7 active compounds against EV-A71 infection. (**A**) Venn diagram showing the intersection of disease genes, drug targets, and core targets. (**B**) Compound–target–disease network. The diamond nodes represent 7 active compounds (lycorine, berberine, matrine, emetine, cepharanthine, oxysophocarpine, harmine), the circular nodes represent the target genes, and the square node represents EV-A71 infection. (**C**) Target–pathway network. The circular nodes represent the core target genes, and the rectangular nodes represent the key KEGG signaling pathways. (**D**) Sankey diagram visualizing the flow relationship between compounds, core targets, and key pathways. (**E**) Venn diagram of the intersection of 4 centrality metrics (MCC, BC, CC_net, DC) for screening core targets from the PPI network. (**F**) GO enrichment analysis of the 155 intersection targets. The bar length represents the −log10 (*p*-value), and the color indicates the GO category (BP: biological process, CC_GO: cellular component, MF: molecular function). (**G**) PPI network of the core targets. The node size is proportional to the degree value, and the edge color represents the interaction confidence. (**H**) KEGG pathway enrichment analysis of the 155 intersection targets. The bubble size represents the number of enriched genes, and the color represents the −log10 (*p*-value).

Building on the meta-analysis findings suggesting potential the anti-EV-A71 efficacy of these seven alkaloids, network pharmacology analysis was performed to explore the potential multi-target mechanisms underlying their observed intervention effects. A total of 1768 EV-A71-related disease targets were obtained from the GeneCards database, and 444 compound-related targets were predicted via SwissTargetPrediction. The intersection analysis yielded 155 common drug-disease targets, which were visualized using a Venn diagram (Figure 9A).

A compound–target–disease network was constructed to illustrate the multi-component, multi-target regulatory relationship, with 7 compounds connected to 155 common targets and the central node of EV-A71 infection (Figure 9B). Subsequently, a target-pathway network was established to link the core targets with key signaling pathways, providing a preliminary overview of the functional pathways involved (Figure 9C). A Sankey diagram was further generated to visualize the direct flow relationship between the 7 compounds, 4 core targets (JUN, MAPK1, MAPK3, AURKB), and 10 key pathways, highlighting the core regulatory axes (Figure 9D).

To screen hub targets from the PPI network, 4 centrality metrics (MCC, BC, CC_net, DC) were calculated, and their intersection was used to identify core targets (Figure 9E). The intersection of four centrality algorithms identified five core targets (MAPK1, MAPK3, JUN, AURKB, and MAPK8; Table 4). The PPI network of core targets was constructed and visualized, with node size proportional to the degree value, revealing the close interactions among hub genes such as MAPK1, MAPK3, and JUN (Figure 9G).

GO enrichment analysis of the 155 intersection targets showed that the biological processes were mainly enriched in immune response, inflammatory response, and cell proliferation, while the molecular functions were primarily related to protein binding and kinase activity (Figure 9F). KEGG pathway enrichment analysis indicated that the core targets were mainly involved in pathways closely associated with viral infection and inflammation, including the MAPK signaling pathway, tumor necrosis factor (TNF) signaling pathway, and interleukin-17 (IL-17) signaling pathway, as well as pathways in cancer (Figure 9H).

### 3.3. Molecular Docking Analysis of Core Compounds and Predicted Targets

To further explore the binding feasibility between the predicted core targets identified by network pharmacology and representative alkaloids, in silico molecular docking analyses were performed. The complete docking results for all assessed pairs are provided in Supplementary Table S11.

**Figure 10:**
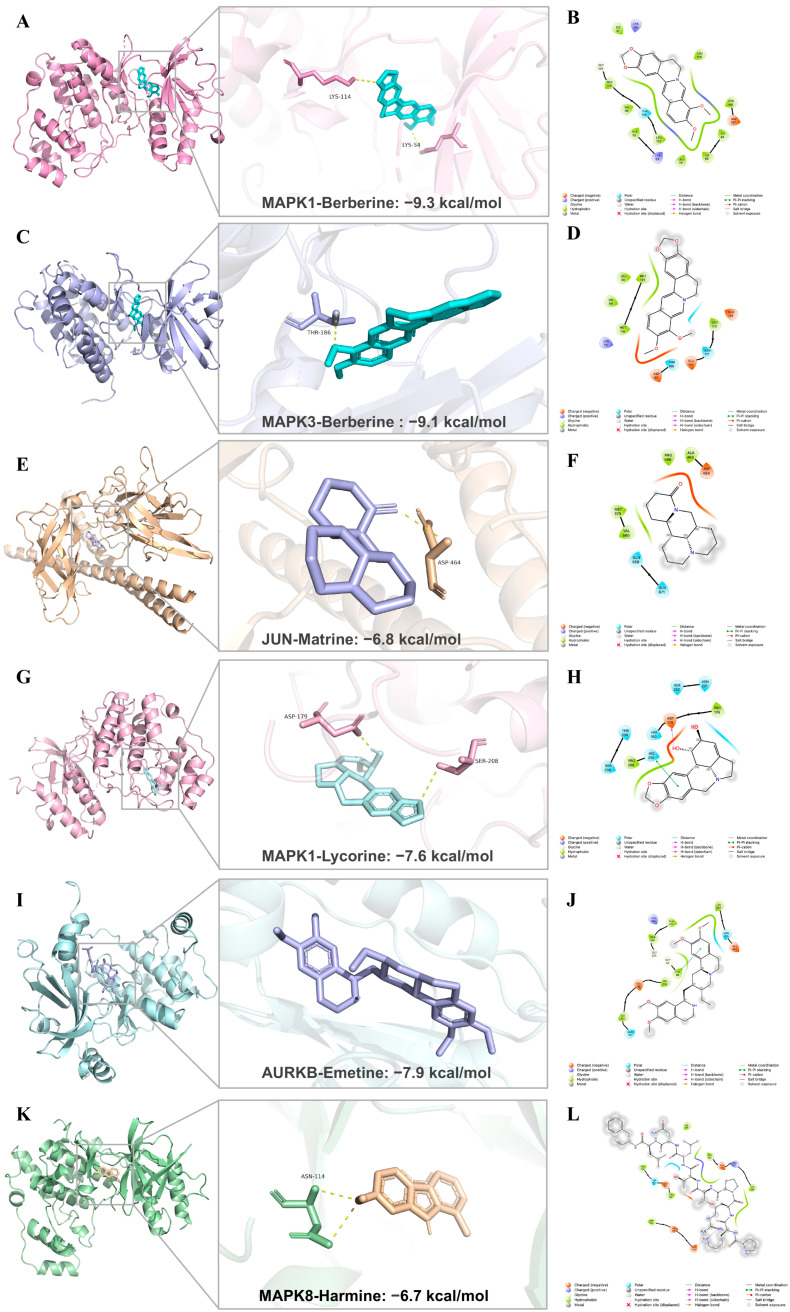
Molecular docking validation of core active compounds with key target proteins. (**A**,**B**) 3D binding mode and 2D interaction diagram of berberine with MAPK1 (binding affinity: −9.3 kcal/mol). (**C**,**D**) 3D binding mode and 2D interaction diagram of berberine with MAPK3 (binding affinity: −9.1 kcal/mol). (**E**,**F**) 3D binding mode and 2D interaction diagram of matrine with JUN (binding affinity: −6.8 kcal/mol). (**G**,**H**) 3D binding mode and 2D interaction diagram of lycorine with MAPK1 (binding affinity: −7.6 kcal/mol). (**I**,**J**) 3D binding mode and 2D interaction diagram of emetine with AURKB (binding affinity: −7.9 kcal/mol). (**K**,**L**) 3D binding mode and 2D interaction diagram of harmine with MAPK8 (binding affinity: −6.7 kcal/mol). All the 2D diagrams illustrate detailed non-covalent interactions.

Berberine exhibited the strongest binding affinity with MAPK1 at −9.3 kcal/mol. It formed stable hydrogen bonds with key residues LYS-54 and LYS-114 in the active pocket, accompanied by extensive hydrophobic interactions (Figure 10A,B). For MAPK3, berberine displayed a binding affinity of −9.1 kcal/mol and formed a critical hydrogen bond with THR-185, enabling tight insertion into the binding domain (Figure 10C,D). Matrine bound well with JUN with a binding affinity of −6.8 kcal/mol. It formed a stable hydrogen bond with ASP-464 and matched the hydrophobic cavity of JUN through hydrophobic contacts (Figure 10E,F). Lycorine bound stably to MAPK1 with a binding affinity of −7.6 kcal/mol. It formed hydrogen bonds with ASP-179 and SER-208, which contributed to strong polar interactions within the active site (Figure 10G,H). Emetine bound to AURKB with a binding affinity of −7.9 kcal/mol. It formed multiple hydrogen bonds and hydrophobic interactions with residues in the ATP-binding pocket, supporting stable complex formation (Figure 10I,J). Harmine bound to MAPK8 with a binding affinity of −6.7 kcal/mol, forming a hydrogen bond with ASN-114 and stable hydrophobic interactions, which ensured firm binding (Figure 10K,L). The 2D interaction diagrams revealed that all compounds could form hydrogen bonds and hydrophobic interactions with key amino acid residues in the target protein pockets, providing computational structural support for the predicted binding interactions.

## 4. Discussion

### 4.1. Summary of Evidence

This study is the first to integrate network pharmacology and molecular docking with preclinical meta-analysis to systematically evaluate the intervention effects of alkaloids against EV-A71 infection. The meta-analysis included nine studies, covering nine alkaloids or their derivatives. The core meta-analytic evidence suggested that natural alkaloid intervention significantly improved the survival rate of EV-A71-infected animals (OR = 30.62, 95% CI: 10.44–89.82, *p* < 0.00001), with no heterogeneity observed for this outcome (I^2^ = 0%) and moderate GRADE certainty of evidence. It should be noted that the large pooled odds ratio for survival (OR = 30.62) should be interpreted cautiously. Effect estimates from small animal studies using lethal infection models may be inflated, particularly when zero-event data or near-complete mortality occurs in the control group. Therefore, this result should not be interpreted as indicating a comparable magnitude of benefit in human EV-A71 infection, but rather as evidence of intervention efficacy under specific experimental conditions. Alkaloid intervention also significantly reduced clinical severity scores, alleviated EV-A71-induced body weight loss, and decreased viral loads in brain and hind limb muscle tissues, indicating effective inhibition of viral replication in vivo. Pre-specified subgroup analyses identified a preliminary trend that quinolizidine alkaloids and high-dose regimens (>5 mg/kg) exhibited superior protective effects, providing clear directions for future drug development. Substantial heterogeneity was observed across several secondary outcomes, likely reflecting differences in mouse strains, ages, viral strains, infection models, alkaloid types, dosages, administration routes, and intervention timing. Consequently, these pooled estimates should primarily be regarded as hypothesis-generating rather than definitive evidence. Methodological quality assessment indicated that the included studies were of moderate overall quality, with inadequate reporting of key methodological details such as randomization and blinding, and the certainty of evidence for secondary outcomes was rated as low to moderate. Nevertheless, Orwin’s fail-safe N analysis (*n* = 279) and sensitivity analyses confirmed that the core conclusion that alkaloids improve survival in infected animals is robust and minimally affected by publication bias.

Network pharmacology analysis predicted 155 common targets shared by seven alkaloids and EV-A71 infection. Core targets (MAPK1, MAPK3, JUN, AURKB, MAPK8) were predicted to be enriched in the MAPK signaling pathway, TNF signaling pathway, and IL-17 signaling pathway, which are critically involved in viral replication and host inflammatory responses. In silico molecular docking further predicted stable binding affinities between active compounds and these core targets (ranging from −6.7 to −9.3 kcal/mol), providing computational structural support for the hypothesized compound–target interactions. Although these targets were consistently identified by network pharmacology analysis, they remain computational predictions. Experimental validation using molecular and virological approaches, including gene knockdown, pathway inhibition, protein expression analysis, and viral replication assays, will be required to establish their functional relevance.

### 4.2. Mechanism Overview

#### 4.2.1. Direct Antiviral Mechanisms Targeting the EV-A71 Life Cycle

Several alkaloids demonstrate direct antiviral activity by interfering with distinct stages of the EV-A71 replication cycle. Cepharanthine, a bisbenzylisoquinoline alkaloid, blocks viral entry by neutralizing endolysosomal acidification, thereby sequestering virions within lysosomes and preventing uncoating [30]. This mechanism targets the early phase of infection and operates independently of viral binding or internalization. Emetine inhibits viral replication at the post-entry stage by suppressing IRES-driven translation of the viral polyprotein, directly targeting the viral translation apparatus [47]. Lycorine and its derivatives (native lycorine, 7e, and LY-55) disrupt viral protein synthesis by blocking polyprotein elongation [32]. Furthermore, LY-55 attenuates virus-induced autophagy through the inhibition of JNK phosphorylation, thereby limiting viral replication [34]. Collectively, these direct-acting mechanisms target viral entry, translation, and replication, achieving the potent inhibition of EV-A71.

#### 4.2.2. Host-Directed Protective Mechanisms: Anti-Inflammatory and Antioxidant Effects

Beyond direct antiviral actions, several alkaloids confer host protection by modulating inflammatory responses, oxidative stress, and immune function. Berberine activates the Keap1-Nrf2 antioxidant pathway, reducing reactive oxygen species (ROS) production and alleviating EV-A71-induced neurological damage [33]. Harmine suppresses nuclear factor kappa-light-chain-enhancer of activated B cells (NF-κB) activation and reduces ROS generation, thereby inhibiting viral replication and mitigating inflammatory responses [49]. Both compounds also lower the levels of pro-inflammatory cytokines (tumor necrosis factor-α [TNF-α], interleukin-1β [IL-1β], interleukin-6 [IL-6]) in infected tissues. Matrine and oxysophocarpine, belonging to the quinolizidine class, enhance survival by restoring T-cell populations (CD3^+^, CD4^+^, CD8^+^), thereby bolstering adaptive immunity [50,51]. These host-oriented mechanisms synergize with direct antiviral effects by reducing tissue damage, controlling excessive inflammation, and facilitating immune-mediated viral clearance.

#### 4.2.3. Mechanisms Predicted by Network Pharmacology and Molecular Docking

Network pharmacology analysis predicted that seven alkaloids (berberine, cepharanthine, emetine, harmine, lycorine, matrine, oxysophocarpine) share 155 overlapping targets with EV-A71 infection. Core targets included MAPK1, MAPK3, JUN, AURKB, and MAPK8, which were predicted to be significantly enriched in the MAPK signaling pathway, TNF signaling pathway, and IL-17 signaling pathway.

The MAPK pathway, a key regulator of cell proliferation, differentiation, and inflammation, corroborates the experimental evidence that LY-55 inhibits c-Jun N-terminal kinase (JNK, a MAPK family member) phosphorylation [34]. The TNF and IL-17 pathways are consistent with the observed reduction in pro-inflammatory cytokines following berberine and harmine intervention [33,49]. Molecular docking predicted favorable binding affinities between representative alkaloids and the identified targets, providing computational evidence that these interactions are structurally feasible. However, these findings should be regarded as hypothesis-generating rather than mechanistic validation and require confirmation by experimental studies. Notably, AURKB (aurora kinase B) remains experimentally uncharacterized in the context of EV-A71 infection and constitutes a novel hypothesis for future investigations. The consistency between computational predictions and experimental findings highlights the multi-target, multi-pathway nature of alkaloid-mediated anti-EV-A71 effects.

### 4.3. Strengths and Limitations

This is the first study to integrate network pharmacology analysis with a meta-analysis of preclinical evidence to comprehensively evaluate the intervention effects of alkaloids against EV-A71 infection. This dual approach not only enables a quantitative synthesis of efficacy data, but also facilitates systematic prediction of the underlying multi-target mechanisms, thereby bridging the gap between computational predictions and experimental evidence. The incorporation of molecular docking further explored the binding affinities between active compounds and core targets (MAPK1, MAPK3, JUN, AURKB, MAPK8), providing structural support for the predicted compound–target interactions. This study systematically included 9 eligible animal experiments covering 7 core alkaloids, including isoquinoline, indole, and quinolizidine alkaloids, achieving a comprehensive integration of anti-EV-A71 evidence of different alkaloid subtypes. Pre-specified subgroup analyses based on alkaloid type and dose were performed to explore potential sources of heterogeneity and provide insights into optimal intervention strategies. The study strictly adhered to the PRISMA guidelines, employed the SYRCLE tool and GRADE approach to assess methodological quality and evidence certainty, respectively, and registered the protocol in PROSPERO. In addition, publication bias and sensitivity analyses were conducted for key outcomes, collectively enhancing the robustness and reliability of the findings.

Several limitations of this study should be acknowledged when interpreting the findings. First, the network pharmacology results are computational predictions based on public databases, which only provide reasonable mechanistic hypotheses rather than direct experimental evidence. In addition, the network pharmacology analysis was performed using human target databases, whereas the efficacy evidence synthesized in this review was derived exclusively from mouse models. Although many signaling pathways are evolutionarily conserved between humans and mice, this species mismatch introduces an additional source of indirectness and may influence the biological relevance of the predicted targets. Therefore, these computational predictions should be interpreted cautiously and require experimental verification in appropriate animal and human systems. Moreover, two semi-synthetic lycorine derivatives (7e and LY-55) were excluded from network pharmacology analysis because their chemical structures lack canonical SMILES identifiers in public databases, precluding reliable target prediction. The regulatory effects of the identified core targets and pathways require further verification via in vitro and in vivo functional experiments, and the prediction results are constrained by the integrity and accuracy of the underlying databases. Second, molecular docking results are based on rigid docking simulations and do not fully capture dynamic conformational changes or the influence of cellular environments on binding, which may affect the accuracy of binding affinity predictions. Furthermore, network pharmacology and molecular docking are computational approaches based on available databases and static protein structures. These methods do not account for compound metabolism, pharmacokinetic behavior, protein conformational dynamics, or the complex biological environment in vivo. Therefore, the predicted compound–target interactions should be interpreted as hypotheses for future experimental investigation rather than definitive mechanisms of action. Third, all 9 included studies were preclinical animal experiments with moderate overall methodological quality. Most studies failed to fully report key methodological details such as randomization and blinding, which may lead to overestimation of the effect size, with the overall certainty of evidence rated as low to moderate. Fourth, significant statistical heterogeneity was observed in multiple core outcomes. Although partial sources of heterogeneity were identified via subgroup analysis, the limited number of included studies (*n* = 9) restricted the prespecified subgroup analyses to variables with at least two studies per category (i.e., alkaloid type and dose). Additional subgroup explorations based on animal strain, timing of administration, or viral strain were not feasible due to sparse data. The high unresolved heterogeneity in secondary outcomes means that their pooled estimates are hypothesis-generating rather than definitive. A critical caveat to the pooled analysis is the pharmacological diversity of the alkaloids included. While the subgroup analysis by alkaloid class (isoquinoline vs. quinolizidine) provided preliminary insights, the limited number of studies per individual compound (*n* = 1–4) precluded a robust single-compound meta-analysis. Therefore, the pooled effect size should not be interpreted as evidence that all alkaloids share equivalent efficacy. Rather, it provides an exploratory summary of the overall signal across a diverse group of compounds. Future research should prioritize head-to-head comparisons of different alkaloids under standardized experimental conditions to enable direct efficacy comparisons and identify the most promising lead compounds. Another important source of clinical heterogeneity was the timing of intervention. The included studies comprised prophylactic pre-intervention before viral challenge, early post-infection intervention, and combined pre-intervention/post-intervention protocols. These intervention paradigms represent distinct biological and clinical scenarios. Therefore, the pooled estimates should not be interpreted exclusively as evidence of therapeutic efficacy after symptom onset but rather as an overall synthesis of currently available preclinical intervention studies. Moreover, meta-regression was not performed, as it is generally not recommended when the number of studies is less than 10. Consequently, the limited number of studies precluded a comprehensive exploration of other potential confounding factors, and the observed heterogeneity reduced the certainty of the pooled effect estimates. In addition, the pooled estimates presented in this review should not be interpreted as evidence of a universal class effect of alkaloids. The included compounds belong to different structural subclasses and differ substantially in their antiviral mechanisms, pharmacokinetic characteristics, toxicity profiles, and host–target interactions. Because most individual alkaloids were investigated in only one independent study, compound-specific meta-analysis was not statistically feasible. Accordingly, the pooled estimates should be interpreted as an overall synthesis of currently available preclinical evidence rather than evidence supporting comparable efficacy across individual alkaloids. Future studies evaluating the same alkaloid under standardized experimental conditions will enable compound-specific quantitative synthesis. Fifth, all studies were conducted in mouse models, and the vast majority used intraperitoneal administration, which is significantly different from clinical scenarios, limiting translational relevance. Furthermore, the included studies primarily employed intraperitoneal administration at doses of 10–20 mg/kg, which differ substantially from potential clinical administration routes and may not directly translate to human exposure. Pharmacokinetic characteristics, dose conversion, oral bioavailability, metabolism, and safety profiles require further investigation before clinical translation can be considered. Finally, while our findings suggest the efficacy of alkaloids against EV-A71 infection, the methodological limitations of the included studies underscore the need for more rigorous preclinical investigations to strengthen the evidence for potential clinical translation.

## 5. Conclusions

This study provides the first integrated exploratory evidence combining meta-analysis, network pharmacology, and molecular docking to evaluate the potential intervention effects of alkaloids against EV-A71 infection. The meta-analysis of nine preclinical studies suggested that alkaloid intervention significantly improves survival rate (OR = 30.62), reduces clinical severity, attenuates body weight loss, and decreases viral loads in brain and hind limb muscle tissues, with the primary outcome showing moderate GRADE certainty and robustness against publication bias. Subgroup analyses identified quinolizidine alkaloids (matrine, oxysophocarpine) and high-dose regimens (>5 mg/kg) as more effective in alleviating body weight loss and clinical symptoms. The pooled meta-analytic findings suggest a promising signal of efficacy across a diverse group of alkaloids, but the pharmacological diversity of these compounds precludes a definitive conclusion of a uniform class effect. The results should be interpreted as hypothesis-generating and should inform the selection of specific lead compounds for future investigation.

Based on the existing experimental literature and our computational predictions, we propose the following multi-mechanistic hypothesis for future investigation: direct antiviral effects by targeting viral entry (cepharanthine), IRES-driven translation (emetine), and polyprotein elongation/autophagy (lycorine and its derivatives); and host-directed protective effects via Nrf2-mediated antioxidant pathways (berberine), NF-κB inhibition (harmine), and T-cell restoration (matrine, oxysophocarpine). Network pharmacology provided computational predictions involving 155 common targets, with core targets (MAPK1, MAPK3, JUN, AURKB, MAPK8) predicted to be enriched in the MAPK, TNF, and IL-17 signaling pathways. In silico molecular docking further predicted stable binding affinities between these core targets and active alkaloids (−6.7 to −9.3 kcal/mol), providing computational support for the hypothesized mechanistic axes. These computational predictions collectively generate a coherent mechanistic hypothesis; however, they must be interpreted as hypothesis-generating rather than mechanistic confirmations. Rigorous experimental analysis is essential before any definitive conclusions regarding mechanisms of action can be drawn.

These findings provide several directions for future research. First, it fills the gap in the lack of quantitative systematic reviews on natural products against EV-A71, establishing a standardized methodological framework for future meta-analyses in the field of antiviral natural products. Second, the core targets (MAPK1, MAPK3, MAPK8, JUN) and signaling pathways (MAPK, TNF pathway) predicted in this study provide clear directions for future mechanistic analysis experiments. Third, the differences in efficacy among different alkaloid subclasses offer important clues for the screening and optimization of lead compounds in the development of novel anti-EV-A71 drugs.

## Figures and Tables

**Table 1:** Summary characteristics of the included studies.

Species	EV-A71 Infection Model	Intervention			Control	Outcome	Refs.
		Types of Alkaloids	Dose/ Duration	Administration			
ICR mice (7 days)	EV-A71 MP4 (1 × 10^7^ TCID50, i.p.)	Cepharanthine (bisbenzylisoquinoline alkaloid)	10 mg/kg, pre-EV-A71 (12 h) + post-EV-A71 (q12h × 4)	i.p.	0.05% DMSO vehicle	1. Survival rate 2. Body weight 3. Clinical scores 4. Viral loads in brain, spinal cord, hind limb muscle 5. Histopathological lesions in brain and muscle	[30]
ICR newborn mice (within 24 h)	EV-A71 C4 (1 × 10^6^ TCID50, i.p.)	Berberine (isoquinoline alkaloid)	5 mg/kg, post-EV-A71 (qd × 7, initiated at 2 h)	i.p.	10% DMSO + 40% Propylene Glycol in PBS	1. Survival rate 2. Body weight 3. clinical scores 4. Viral loads in brain, lung, heart, liver, kidney 5. EV-A71 VP1 protein expression in brain, lung, heart, liver, kidney 6. Histopathological injury of brainstem and lung 7. Pro-inflammatory cytokine (TNF-α, IL-1β, IL-6) levels in brain and lung tissues	[33]
ICR mice (10 days)	EV-A71-MP10 (1 × 10^7^ TCID50, i.p.)	Lycorine derivative 7e (amaryllidaceae alkaloid)	1 mg/kg, post-EV-A71 (qd × 6)	i.p.	Saline	1. Survival rate 2. Clinical scores	[48]
KM mice (2 weeks)	EV-A71 GZ-CII strain (1.2 × 10^8^ PFU, i.p.)	Emetine (isoquinoline alkaloid)	0.20 mg/kg, pre-EV-A71 (6 h) + post-EV-A71 (q12h × 8)	i.g.	40% hydroxypropyl-beta-cyclodextrin vehicle	1. Survival rate 2. Clinical scores 3. Viral loads in fore limbs, hind limbs, brain and spleen	[47]
ICR suckling mice (12 days)	EV-A71-H-MA (10 LD_50_, i.p.)	Lycorine derivative LY-55 (isoquinoline alkaloid)	1.5 mg/kg, post-EV-A71 (qd × 7)	i.p.	Sterile water	1. Survival rate 2. Body weight 3. Clinical scores 4. Viral loads in hind limb 5. EV-A71 VP1 protein expression in muscle 6. Muscle histopathological injury scores	[34]
AG129 mice (5 weeks)	Gluc-EV-A71 (2 × 10^6^ TCID50, i.p.)	Harmine (β-carboline alkaloid)	12.5 mg/kg, post-EV-A71 (qd × 4)	i.p.	1 × PBS	1. Viral loads 2. Viral replication bioluminescence signals in thoracic cavity and intestinal tract 3. Viral replication level in intestinal tissues	[49]
BALB/c mice (3 months)	Lethal EV-A71 infection model (i.p.)	Oxysophocarpine (quinolizidine alkaloid)	15 mg/kg, post-EV-A71 (qd × 14)	i.p.	Placebo	1. Survival rate 2. Body weight 3. Clinical scores 4. Viral loads in skeletal muscle 5. CD3^+^/CD4^+^/CD8^+^ T lymphocyte counts	[50]
ICR mice (10 days)	EV-A71-MP10 (1 × 10^7^ TCID50, i.p.)	Matrine (quinolizidine alkaloid)	20 mg/kg, post-EV-A71 (qd × 6)	i.p.	Saline	1. Survival rate 2. Clinical scores 3. Viral loads in hind limb	[51]
ICR mice (10–11 days)	EV-A71-MP10 (1 × 10^7^ TCID50, i.p.)	Lycorine (Amaryllidaceae alkaloid)	0.4 mg/kg, post-EV-A71 (bid × 7, initiated at 12 h)	i.p.	Saline	1. Survival rate 2. Clinical scores 3. Viral loads in hind limb 4. Paralysis incidence 5. Pathological damage degree of skeletal muscle	[32]

bid, bis in die; CD3^+^, cluster of differentiation 3 positive; CD4^+^, cluster of differentiation 4 positive; CD8^+^, cluster of differentiation 8 positive; DMSO, dimethyl sulfoxide; EV-A71, enterovirus A71; i.g., intragastric administration; i.p., intraperitoneal injection; IL-1β, interleukin-1β; IL-6, interleukin-6; LD_50_, 50% lethal dose; PBS, phosphate-buffered saline; PFU, plaque forming unit; q12h, quaque 12 hora; qd, quaque die; TCID50, 50% tissue culture infectious dose; TNF-α, tumor necrosis factor-α; VP1, viral protein 1.

**Table 2:** Basic characteristics of the included alkaloids.

Compound	Chemical Structure	Chemical Class	Subclass	Plant Source	CAS Number	Molecular Formula	Molecular Weight	Refs.
Cepharanthine	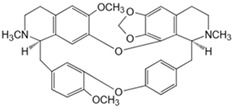	Isoquinoline	Bisbenzylisoquinoline	*Stephania cepharantha* (Menispermaceae), tuber	481-49-2	C_37_H_38_N_2_O_6_	606.71	[30]
Berberine	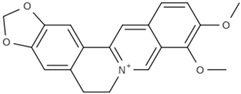	Isoquinoline	Protoberberine	*Coptis chinensis* (Ranunculaceae), rhizome	2086-83-1	C_20_H_18_NO_4_^+^	336.36	[33]
Lycorine derivative 7e	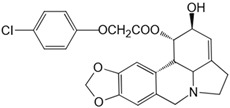	Isoquinoline	Lycorine-type (semi-synthetic)	Semi-synthetic derivative of lycorine (from *Lycoris radiata*, Amaryllidaceae)	-	C_24_H_23_ClNO_6_	456.90	[48]
Emetine	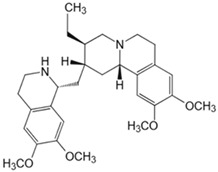	Isoquinoline	Emetine-type	*Carapichea ipecacuanha* (Rubiaceae), root	483-18-1	C_29_H_40_N_2_O_4_	480.60	[47]
Lycorine derivative LY-55	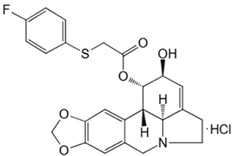	Isoquinoline	Lycorine-type (semi-synthetic)	Semi-synthetic derivative of lycorine (from *Lycoris radiata*, Amaryllidaceae)	-	C_24_H_22_FNO_5_S·HCl	492.00	[34]
Harmine	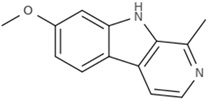	Indole	β-Carboline	*Peganum harmala* (Nitrariaceae), seed	442-51-3	C_13_H_12_N_2_O	212.25	[49]
Oxysophocarpine	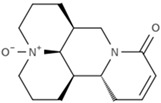	Quinolizidine	Matrine-type	*Sophora alopecuroides* (Fabaceae), root	26904-64-3	C_15_H_22_N_2_O_2_	262.35	[50]
Matrine	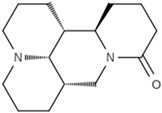	Quinolizidine	Matrine-type	*Sophora flavescens* (Fabaceae), root	519-02-8	C_15_H_24_N_2_O	248.36	[51]
Lycorine	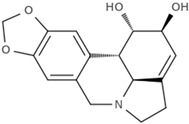	Isoquinoline	Lycorine-type	*Lycoris radiata* (Amaryllidaceae), bulb	476-28-8	C_16_H_17_NO_4_	287.31	[32]

Oxysophocarpine is classified as a quinolizidine alkaloid because it is an oxidized derivative of sophocarpine that retains the characteristic quinolizidine skeleton despite oxidation.

**Table 3:** Orwin’s fail-safe N analysis for survival rate.

**Classic Fail-Safe N**	
Z-value for observed studies	6.23
The *p*-value for observed studies	0.00
Alpha	0.05
Tails	2.00
Z for alpha	1.96
Number of observed studies	8.00
Number of missing studies that would bring *p*-value to >alpha	73.00
**Orwin’s Fail-Safe N**	
The odds ratio in observed studies	30.62
The criterion for a ‘trivial’ odds ratio	1.100
Mean odds ratio in missing studies	1.000
Number missing studies needed to bring odds ratio under 1.1	279.00

Alpha, statistical significance level (Type I error probability); N, number of studies; OR, odds ratio; *p*, probability value; Z, Z-score (standard normal deviate).

**Table 4:** Core targets of the nine compounds identified by network pharmacology analysis.

Symbol	Gene	Degree
MAPK1	Mitogen-activated protein kinase 1	2
MAPK3	Mitogen-activated protein kinase 3	2
JUN	Transcription factor AP-1	1
AURKB	Aurora kinase B	1
MAPK8	Mitogen-activated protein kinase 8	1

Note: Degree values represent the number of active compounds targeting each gene in the compound–target interaction network, not the number of protein–protein interactions.

## Data Availability

Data are contained within the article and Supplementary Materials. All data generated or analyzed during this study are included in this published article.

## Supplementary Materials

The following supporting information can be downloaded at: https://www.mdpi.com/article/10.3390/ijms27156571/s1.
