# Supplementary material for: Efficacy and Mechanisms of Alkaloids Against Enterovirus A71: A Systematic Review and Meta-Analysis of Preclinical Evidence Integrated with Network Pharmacology and Molecular Docking
Source: Int J Mol Sci. 2026 Jul 23;27(15):6571. doi: 10.3390/ijms27156571 (PMC13465392; doi:10.3390/ijms27156571)
Supplement: Supplementary file 1 [file ijms-27-06571-s001.zip › Supplementary Materials.pdf]

Supplementary Material

Table S1. Data sources for systematic review.

Resource	Full Name	Version / Access Date	Description	Peer-Reviewed	International/ Local	Language	Purpose in This Study
**PubMed**	PubMed	Accessed on 2 Mar 2026	Biomedical literature database maintained by US National Center for Biotechnology Information (NCBI)	Yes	International	English	Literature retrieval for systematic review
**Embase**	Excerpta Medica database	Accessed on 2 Mar 2026	Biomedical and pharmacological database maintained by Elsevier	Yes	International	English	Comprehensive literature retrieval
**Cochrane Library**	Cochrane Central Register of Controlled Trials	Accessed on 2 Mar 2026	Specialized trials register part of Cochrane Library	Yes	International	English	Identification of randomized controlled trials
**Web of Science**	Web of Science Core Collection	Accessed on 2 Mar 2026	Multidisciplinary academic literature and citation database maintained by Clarivate Analytics	Yes	International	English	Multidisciplinary literature retrieval for systematic review
**Scopus**	Scopus Abstract and Citation Database	Accessed on 2 Mar 2026	Multidisciplinary abstract and citation database maintained by Elsevier	Yes	International	English	Comprehensive multidisciplinary literature retrieval
**CNKI**	China National Knowledge	Accessed on 2 Mar 2026	Comprehensive Chinese academic resource hosting journals, theses	Yes (hosted	Local (China)	Primarily Chinese	Retrieval of Chinese literature

Resource	Full Name	Version / Access Date	Description	Peer-Reviewed	International/ Local	Language	Purpose in This Study
	Infrastructure		and conference proceedings	content)			
**Wanfang**	Wanfang Data	Accessed on 2 Mar 2026	Chinese knowledge service platform providing scholarly journals, dissertations and patents	Yes (hosted content)	Local (China)	Primarily Chinese	Retrieval of Chinese literature
**VIP**	VIP Chinese Scientific Journals Database	Accessed on 2 Mar 2026	Full-text database of Chinese academic journals	Yes (hosted content)	Local (China)	Primarily Chinese	Retrieval of Chinese literature

Table S2. The detailed search strategy.

Electronic databases	Search	Search strategy	Results
**PubMed**	#1	(((((((((((("Alkaloids"[Mesh]) OR (alkaloid agent[Title/Abstract])) OR (alkaloid mixture[Title/Abstract])) OR (alkaloid, phenolic[Title/Abstract])) OR (apocynaceae alkaloid[Title/Abstract])) OR (heterocyclic alkaloid[Title/Abstract])) OR (phenolic alkaloid[Title/Abstract])) OR (roacanga alkaloid[Title/Abstract])) OR (Alkaloid[Title/Abstract])) OR (Plant Alkaloids[Title/Abstract])) OR (Alkaloids, Plant[Title/Abstract])) OR (Plant Alkaloid[Title/Abstract])) OR (Alkaloid, Plant[Title/Abstract])	529.958
	#2	((((((((((("Enterovirus A, Human"[Mesh]) OR (Human Enterovirus A[Title/Abstract])) OR (Enterovirus 71, Human[Title/Abstract])) OR (Enterovirus A71[Title/Abstract])) OR (EV-A71[Title/Abstract])) OR (EV A71[Title/Abstract])) OR (EVA71[Title/Abstract])) OR (Human Enterovirus 71[Title/Abstract])) OR (Enterovirus 71[Title/Abstract])) OR (EV71[Title/Abstract])) OR (EV 71[Title/Abstract])) OR (Enterovirus A[Title/Abstract])	4,135
	#3	#1 AND #2	**25**
**Embase**	#1	'alkaloid'/exp OR alkaloid	818,516

	#2	'alkaloid agent':ab,ti OR 'alkaloid mixture':ab,ti OR 'alkaloid, phenolic':ab,ti OR 'apocynaceae alkaloid':ab,ti OR 'heterocyclic alkaloid':ab,ti OR 'phenolic alkaloid':ab,ti OR 'roacanga alkaloid':ab,ti OR 'alkaloid':ab,ti OR 'plant alkaloids':ab,ti OR 'alkaloids, plant':ab,ti OR 'plant alkaloid':ab,ti OR 'alkaloid, plant':ab,ti OR 'alkaloids':ab,ti	80,182
	#3	#1 OR #2	827,429
	#4	('enterovirus'/exp OR enterovirus) AND 71	4,764
	#5	'enterovirus a, human':ab,ti OR 'human enterovirus a':ab,ti OR 'enterovirus 71, human':ab,ti OR 'enterovirus a71':ab,ti OR 'ev-a71':ab,ti OR 'ev a71':ab,ti OR 'eva71':ab,ti OR 'human enterovirus 71':ab,ti OR 'enterovirus 71':ab,ti OR 'ev71':ab,ti OR 'ev 71':ab,ti OR 'enterovirus a':ab,ti	4,506
	#6	#4 OR #5	6,084
	#7	#3 AND #6	**84**
**Cochrane Library**	#1	MeSH descriptor: [Alkaloids] explode all trees	41,402
	#2	(alkaloid agent or alkaloid mixture or alkaloid, phenolic or apocynaceae alkaloid or heterocyclic alkaloid or phenolic alkaloid or roacanga alkaloid or Alkaloid or Plant Alkaloids or Alkaloids, Plant or Plant Alkaloid or Alkaloid, Plant):ti,ab,kw	439
	#3	#1 or #2	41,637
	#4	MeSH descriptor: [Enterovirus A, Human] explode all trees	43
	#5	(Human Enterovirus A or Enterovirus 71, Human or Enterovirus A71 or EV-A71 or EV A71 or EVA71 or Human Enterovirus 71 or Enterovirus 71 or EV71 or EV 71 or Enterovirus A):ti,ab,kw	331
	#6	#4 or #5	331
	#7	#3 and #6	**1**
**Web of Science**	#1	TS=('Alkaloids') OR AB=('alkaloid agent' OR 'alkaloid mixture' OR 'alkaloid, phenolic' OR 'apocynaceae alkaloid' OR 'heterocyclic alkaloid' OR 'phenolic alkaloid' OR 'roacanga alkaloid' OR 'Alkaloid' OR 'Plant Alkaloids' OR 'Alkaloids, Plant' OR 'Plant Alkaloid' OR 'Alkaloid, Plant ')	174,868

	#2	TS=('Enterovirus A, Human') OR AB=('Human Enterovirus A' OR 'Enterovirus 71, Human' OR 'Enterovirus A71' OR 'EV-A71' OR 'EV A71' OR 'EVA71' OR 'Human Enterovirus 71' OR 'Enterovirus 71' OR 'EV71' OR 'EV 71' OR 'Enterovirus A')	36,096
	#3	(#1) AND (#2)	**68**
**Scopus**	#1	TITLE-ABS-KEY ("alkaloid agent" OR "alkaloid mixture" OR "alkaloid, phenolic" OR "apocynaceae alkaloid" OR "heterocyclic alkaloid" OR "phenolic alkaloid" OR "roacanga alkaloid" OR "Alkaloid" OR "Plant Alkaloids" OR "Alkaloids, Plant" OR "Plant Alkaloid" OR "Alkaloid, Plant")	296,511
	#2	TITLE-ABS-KEY ("Enterovirus A, Human" OR "Human Enterovirus A" OR "Enterovirus 71, Human" OR "Enterovirus A71" OR "EV-A71" OR "EV A71" OR "EVA71" OR "Human Enterovirus 71" OR "Enterovirus 71" OR "EV71" OR "EV 71" OR "Enterovirus A")	5,299
	#3	(#1) AND (#2)	**38**
**CNKI**	1#	Title, Keyword and Abstract: 生物碱 + 植物生物碱 + 酚性生物碱 + 杂环生物碱 + 夹竹桃科生物碱	50,803
	2#	Title, Keyword and Abstract: 肠道病毒 71 型 + EV71 + 人肠道病毒 71 型 + 人肠道病毒 A 型 + 肠道病毒 A 型 + EV-A71	7,929
	3#	1# AND 2#	**5**
**WanFang**	1#	All field: 生物碱 OR 植物生物碱 OR 酚性生物碱 OR 杂环生物碱 OR 夹竹桃科生物碱	29,619
	2#	All field: 肠道病毒 71 型 OR EV71 OR 人肠道病毒 71 型 OR 人肠道病毒 A 型 OR 肠道病毒 A 型 OR EV-A71	8,736
	3#	1# AND 2#	**3**
**VIP**	1#	M=("生物碱" OR "植物生物碱" OR "酚性生物碱" OR "杂环生物碱" OR "夹竹桃科生物碱")	327
	2#	M=("肠道病毒 71 型" OR "EV71" OR "人肠道病毒 71 型" OR "人肠道病毒 A 型" OR "肠道病毒 A 型" OR "EV-A71")	4,927
	3#	1# AND 2#	**3**

Table S3. Information of the seven compounds.

Compound Name	PubChem CID	Canonical SMILES	Number of Predicted Targets
**Berberine**	2353	COC1=C(C2=C[N+]3=C(C=C2C=C1)C4=CC5=C(C=C4CC3)OCO5)OC	105
**Cepharanthine**	10206	CN1CCC2=CC3=C(C4=C2[C@@H]1CC5=CC=C(C=C5)OC6=C(C=CC(=C6)C[C@@H]7C8=CC(=C(C=C8CCN7C)OC)O4)OC)OCO3	102
**Emetine**	10219	CC[C@H]1CN2CCC3=CC(=C(C=C3[C@@H]2C[C@@H]1C[C@@H]4C5=CC(=C(C=C5CCN4)OC)OC)OC)OC	130
**Harmine**	5280953	CC1=NC=CC2=C1NC3=C2C=CC(=C3)OC	104
**Lycorine**	72378	C1CN2CC3=CC4=C(C=C3[C@H]5[C@H]2C1=C[C@@H]([C@H]5O)O)OCO4	102
**Matrine**	91466	C1C[C@@H]2[C@H]3CCCN4[C@H]3[C@@H](CCC4)CN2C(=O)C1	101
**Oxysophocarpine**	12309166	C1C[C@H]2CN3[C@H](CC=CC3=O)[C@@H]4[C@H]2[N+](C1)(CCC4)[O-]	52

Table S4. Information of disease target databases and search strategies.

Database	Search Terms	Screening Criteria	Search Date	Number of Targets
**DisGeNET**	Enterovirus A71	score ≥ 0.2	March 2026	26
**GeneCards**	Enterovirus A71	Relevance score ≥ 5	March 2026	109
**GeneCards**	Enterovirus infection	Relevance score ≥ 5	March 2026	4483
**GeneCards**	Hand, foot and mouth disease	Relevance score ≥ 5	March 2026	21
**Combined (after deduplication)**	-	-	-	1768

Table S5. The 155 common targets of compounds and EV71 infection.

Symbol	Description	Degree	ENTREZID	IsCore
**BCHE**	butyrylcholinesterase	6	590	No

**ACHE**	acetylcholinesterase (Yt blood group)	5	43	No
**NOS1**	nitric oxide synthase 1	4	4842	No
**SLC6A3**	solute carrier family 6 member 3	4	6531	No
**CSF1R**	colony stimulating factor 1 receptor	3	1436	No
**CTSK**	cathepsin K	3	1513	No
**F3**	coagulation factor III, tissue factor	3	2152	No
**JAK1**	Janus kinase 1	3	3716	No
**JAK2**	Janus kinase 2	3	3717	No
**ADORA2A**	adenosine A2a receptor	2	135	No
**ADRB2**	adrenoceptor beta 2	2	154	No
**AURKA**	aurora kinase A	2	6790	No
**BRD4**	bromodomain containing 4	2	23476	No
**CDC42**	cell division cycle 42	2	998	No
**CDK1**	cyclin dependent kinase 1	2	983	No
**CDK2**	cyclin dependent kinase 2	2	1017	No
**CDK4**	cyclin dependent kinase 4	2	1019	No
**CDK9**	cyclin dependent kinase 9	2	1025	No
**CHEK2**	checkpoint kinase 2	2	11200	No
**EGFR**	epidermal growth factor receptor	2	1956	No
**EHMT2**	euchromatic histone lysine methyltransferase 2	2	10919	No
**EPHX1**	epoxide hydrolase 1	2	2052	No
**HDAC1**	histone deacetylase 1	2	3065	No
**HDAC2**	histone deacetylase 2	2	3066	No
**ICAM1**	intercellular adhesion molecule 1	2	3383	No
**IKBKB**	inhibitor of nuclear factor kappa B kinase subunit beta	2	3551	No
**IRAK4**	interleukin 1 receptor associated kinase 4	2	51135	No
**JAK3**	Janus kinase 3	2	3718	No
**KCNH2**	potassium voltage-gated channel subfamily H member 2	2	3757	No
**LCK**	LCK proto-oncogene, Src family tyrosine kinase	2	3932	No
**LRRK2**	leucine rich repeat kinase 2	2	120892	No
**MAOA**	monoamine oxidase A	2	4128	No
**MAPK1**	mitogen-activated protein kinase 1	2	5594	Yes
**MAPK14**	mitogen-activated protein kinase 14	2	1432	No
**MET**	MET proto-oncogene, receptor tyrosine kinase	2	4233	No
**MMP1**	matrix metallopeptidase 1	2	4312	No
**MMP13**	matrix metallopeptidase 13	2	4322	No
**NOS2**	nitric oxide synthase 2	2	4843	No

**NOS3**	nitric oxide synthase 3	2	4846	No
**PARP1**	poly(ADP-ribose) polymerase 1	2	142	No
**PRKACA**	protein kinase cAMP-activated catalytic subunit alpha	2	5566	No
**PTGER2**	prostaglandin E receptor 2	2	5732	No
**ROCK1**	Rho associated coiled-coil containing protein kinase 1	2	6093	No
**RPS6KB1**	ribosomal protein S6 kinase B1	2	6198	No
**SELE**	selectin E	2	6401	No
**SIRT2**	sirtuin 2	2	22933	No
**SRC**	SRC proto-oncogene, non-receptor tyrosine kinase	2	6714	No
**TLR9**	toll like receptor 9	2	54106	No
**ABCB1**	ATP binding cassette subfamily B member 1	1	5243	No
**ABCC1**	ATP binding cassette subfamily C member 1 (ABCC1 blood group)	1	4363	No
**ABL1**	ABL proto-oncogene 1, non-receptor tyrosine kinase	1	25	No
**ACVRL1**	activin A receptor like type 1	1	94	No
**AKT1**	AKT serine/threonine kinase 1	1	207	No
**AKT2**	AKT serine/threonine kinase 2	1	208	No
**AKT3**	AKT serine/threonine kinase 3	1	10000	No
**ALDH2**	aldehyde dehydrogenase 2 family member	1	217	No
**ALK**	ALK receptor tyrosine kinase	1	238	No
**ATR**	ATR checkpoint kinase	1	545	No
**BAD**	BCL2 associated agonist of cell death	1	572	No
**BRAF**	B-Raf proto-oncogene, serine/threonine kinase	1	673	No
**BTK**	Bruton tyrosine kinase	1	695	No
**CCR2**	C-C motif chemokine receptor 2	1	729230	No
**CCR5**	C-C motif chemokine receptor 5	1	1234	No
**CD38**	CD38 molecule	1	952	No
**CFD**	complement factor D	1	1675	No
**CHUK**	component of inhibitor of nuclear factor kappa B kinase complex	1	1147	No
**CSNK1A1**	casein kinase 1 alpha 1	1	1452	No
**CSNK2A1**	casein kinase 2 alpha 1	1	1457	No
**CSNK2A2**	casein kinase 2 alpha 2	1	1459	No
**CTSL**	cathepsin L	1	1514	No
**CXCR3**	C-X-C motif chemokine receptor 3	1	2833	No
**CYP1A2**	cytochrome P450 family 1 subfamily A member 2	1	1544	No
**CYP2C19**	cytochrome P450 family 2 subfamily C member 19	1	1557	No
**CYP2C9**	cytochrome P450 family 2 subfamily C member 9	1	1559	No

**CYP2D6**	cytochrome P450 family 2 subfamily D member 6 (gene/pseudogene)	1	1565	No
**DHFR**	dihydrofolate reductase	1	1719	No
**ELANE**	elastase, neutrophil expressed	1	1991	No
**ERAP1**	endoplasmic reticulum aminopeptidase 1	1	51752	No
**ERBB2**	erb-b2 receptor tyrosine kinase 2	1	2064	No
**ERG**	ETS transcription factor ERG	1	2078	No
**ERN1**	endoplasmic reticulum to nucleus signaling 1	1	2081	No
**ESR1**	estrogen receptor 1	1	2099	No
**F2**	coagulation factor II, thrombin	1	2147	No
**FLT1**	fms related receptor tyrosine kinase 1	1	2321	No
**FLT3**	fms related receptor tyrosine kinase 3	1	2322	No
**FUCA1**	alpha-L-fucosidase 1	1	2517	No
**FYN**	FYN proto-oncogene, Src family tyrosine kinase	1	2534	No
**GCK**	glucokinase	1	2645	No
**GLB1**	galactosidase beta 1	1	2720	No
**GSR**	glutathione-disulfide reductase	1	2936	No
**HDAC6**	histone deacetylase 6	1	10013	No
**HDAC8**	histone deacetylase 8	1	55869	No
**HMOX1**	heme oxygenase 1	1	3162	No
**HSP90AA1**	heat shock protein 90 alpha family class A member 1	1	3320	No
**HSPA5**	heat shock protein family A (Hsp70) member 5	1	3309	No
**HSPA8**	heat shock protein family A (Hsp70) member 8	1	3312	No
**IDH1**	isocitrate dehydrogenase (NADP(+)) 1	1	3417	No
**IGF1R**	insulin like growth factor 1 receptor	1	3480	No
**INSR**	insulin receptor	1	3643	No
**JUN**	Jun proto-oncogene, AP-1 transcription factor subunit	1	3725	Yes
**KDM1A**	lysine demethylase 1A	1	23028	No
**KDM5C**	lysine demethylase 5C	1	8242	No
**KDR**	kinase insert domain receptor	1	3791	No
**KIT**	KIT proto-oncogene, receptor tyrosine kinase	1	3815	No
**LIMK1**	LIM domain kinase 1	1	3984	No
**LYN**	LYN proto-oncogene, Src family tyrosine kinase	1	4067	No
**MALT1**	MALT1 paracaspase	1	10892	No
**MAN1B1**	mannosidase alpha class 1B member 1	1	11253	No
**MAP2K1**	mitogen-activated protein kinase kinase 1	1	5604	No
**MAPK10**	mitogen-activated protein kinase 10	1	5602	No
**MAPK3**	mitogen-activated protein kinase 3	1	5595	Yes

**MAPK8**	mitogen-activated protein kinase 8	1	5599	Yes
**MDM2**	MDM2 proto-oncogene	1	4193	No
**MECP2**	methyl-CpG binding protein 2	1	4204	No
**MME**	membrane metalloendopeptidase	1	4311	No
**MMP3**	matrix metallopeptidase 3	1	4314	No
**MMP7**	matrix metallopeptidase 7	1	4316	No
**MMP8**	matrix metallopeptidase 8	1	4317	No
**MPO**	myeloperoxidase	1	4353	No
**MTOR**	mechanistic target of rapamycin kinase	1	2475	No
**NQO1**	NAD(P)H quinone dehydrogenase 1	1	1728	No
**NR3C1**	nuclear receptor subfamily 3 group C member 1	1	2908	No
**PCNA**	proliferating cell nuclear antigen	1	5111	No
**PDPK1**	3-phosphoinositide dependent protein kinase 1	1	5170	No
**PIK3CA**	phosphatidylinositol-4,5-bisphosphate 3-kinase catalytic subunit alpha	1	5290	No
**PIK3CB**	phosphatidylinositol-4,5-bisphosphate 3-kinase catalytic subunit beta	1	5291	No
**PIK3CD**	phosphatidylinositol-4,5-bisphosphate 3-kinase catalytic subunit delta	1	5293	No
**PIK3CG**	phosphatidylinositol-4,5-bisphosphate 3-kinase catalytic subunit gamma	1	5294	No
**PLA2G2A**	phospholipase A2 group IIA	1	5320	No
**PRF1**	perforin 1	1	5551	No
**PRKCA**	protein kinase C alpha	1	5578	No
**PRKCD**	protein kinase C delta	1	5580	No
**PRNP**	prion protein (Kanno blood group)	1	5621	No
**PTGS2**	prostaglandin-endoperoxide synthase 2	1	5743	No
**PTK2**	protein tyrosine kinase 2	1	5747	No
**PTPN11**	protein tyrosine phosphatase non-receptor type 11	1	5781	No
**PTPRC**	protein tyrosine phosphatase receptor type C	1	5788	No
**RAC1**	Rac family small GTPase 1	1	5879	No
**REN**	renin	1	5972	No
**S1PR1**	sphingosine-1-phosphate receptor 1	1	1901	No
**SCARB1**	scavenger receptor class B member 1	1	949	No
**SHH**	sonic hedgehog signaling molecule	1	6469	No
**SIRT1**	sirtuin 1	1	23411	No
**SLC5A1**	solute carrier family 5 member 1	1	6523	No
**SLC5A2**	solute carrier family 5 member 2	1	6524	No
**SLC6A4**	solute carrier family 6 member 4	1	6532	No
**TERT**	telomerase reverse transcriptase	1	7015	No

**TGFBR1**	transforming growth factor beta receptor 1	1	7046	No
**TRPV1**	transient receptor potential cation channel subfamily V member 1	1	7442	No
**TSPO**	translocator protein	1	706	No
**TTR**	transthyretin	1	7276	No
**TYK2**	tyrosine kinase 2	1	7297	No
**TYMS**	thymidylate synthetase	1	7298	No
**TYRO3**	TYRO3 protein tyrosine kinase	1	7301	No
**XBP1**	X-box binding protein 1	1	7494	No

Table S6. Topological parameters of the PPI network. Degree, Betweenness, Closeness, and MCC were calculated using the CytoHubba plugin in Cytoscape. The top 5 genes ranked by Degree centrality (ACHE, ADORA2A, SLC6A3, JAK2, JAK1) were defined as core targets (IsCore = Yes).

Gene	Degree	Betweenness	Closeness	MCC	IsCore
**ACHE**	2.045	0	0.75	0.30779	Yes
**ADORA2A**	2.044	0	0.75	0.30779	Yes
**SLC6A3**	1.901	0	0.75	0.61557	Yes
**JAK2**	1.598	0	0.75	0	Yes
**JAK1**	1.598	0	0.75	0	Yes
**CTSK**	1	0	0	0	No
**CSF1R**	1	0	0	0	No
**BCHE**	1	0	0	0	No

Table S7. Complete list of significantly enriched KEGG pathways for the 155 common targets (p.adjust < 0.05).

Pathway ID	Pathway Description	Gene_Ratio	Background Ratio	p value	Adjusted p value	q value	Gene Count	Gene Symbols
**hsa01522**	Endocrine resistance	27/147	99/9375	8.71648E-27	2.54521E-24	9.63401E-25	27	6198/5293/5291/5602/5566/6714/1432/1019/1565/3480/572/1956/2064/2475/5290/207/5747/3725/5594/2099/5595/208/10000/673/4193/5599/5604
**hsa05417**	Lipid and atherosclerosis	35/147	216/9375	2.79708E-26	4.08373E-24	1.54575E-24	35	998/3383/6401/5293/5291/5602/7494/3717/6714/3551/1432/5879/572/5578/4067/5170/4314/4312/4846/5290/207/

								5747/3725/5594/5595/3320/208/10000/1147/51135/5599/2081/3312/3309/1559
**hsa05212**	Pancreatic cancer	24/147	77/9375	1.92109 E-25	1.86986 E-23	7.07769 E-24	24	998/6198/5293/5291/5602/3551/1019/5879/572/1956/3716/2064/2475/5290/7046/207/5594/5595/208/10000/673/1147/5599/5604
**hsa05235**	PD-L1 expression and PD-1 checkpoint pathway in cancer	24/147	90/9375	1.30774 E-23	9.54649 E-22	3.61349 E-22	24	6198/5293/5291/3717/3932/3551/1432/238/1956/3716/1457/1459/2475/5290/207/3725/5594/5595/208/10000/5781/1147/5604/54106
**hsa05205**	Proteoglycans in cancer	31/147	204/9375	2.09455 E-22	1.22322 E-20	4.63007 E-21	31	998/6198/4233/5293/5291/6093/5566/6714/1432/5879/3480/5578/5170/1956/2064/2475/3791/5290/207/5747/5594/2099/5595/208/10000/5781/673/4193/6469/5604/1514
**hsa01521**	EGFR tyrosine kinase inhibitor resistance	22/147	80/9375	4.98775 E-22	2.42737 E-20	9.18796 E-21	22	6198/4233/5293/5291/3717/6714/3480/572/5578/1956/3716/2064/2475/3791/5290/207/5594/5595/208/10000/673/5604
**hsa04012**	ErbB signaling pathway	22/147	86/9375	2.89434 E-21	1.20735 E-19	4.57001 E-20	22	6198/5293/5291/5602/6714/25/572/5578/1956/2064/2475/5290/207/5747/3725/5594/5595/208/10000/673/5599/5604
**hsa04933**	AGE-RAGE signaling pathway in diabetic complications	23/147	101/9375	6.15403 E-21	2.24622 E-19	8.50228 E-20	23	998/2152/3383/6401/5293/5291/5602/3717/1432/1019/5879/5580/5578/4846/5290/7046/207/3725/5594/5595/208/10000/5599
**hsa05161**	Hepatitis B	27/147	163/9375	1.40153 E-20	4.18597 E-19	1.58445 E-19	27	5293/5291/5602/3717/6714/3551/1432/1017/572/5578/3716/5290/7046/207/3725/5594/5111/55

								95/208/10000/673/1147/3718/51135/5599/5604/7297
**hsa04151**	PI3K-Akt signaling pathway	37/147	362/9375	1.43355 E-20	4.18597 E-19	1.58445 E-19	37	6198/4233/5293/5291/5294/3717/3815/3551/1017/1019/1436/5879/3480/2322/572/5578/5170/3643/1956/3716/2064/2475/3791/4846/2321/5290/207/5747/5594/5595/3320/208/10000/1147/4193/3718/5604
**hsa04370**	VEGF signaling pathway	19/147	60/9375	1.85804 E-20	4.90102 E-19	1.85511 E-19	19	998/5293/5291/6714/1432/5743/5879/572/5578/3791/4846/5290/207/5747/5594/5595/208/10000/5604
**hsa05215**	Prostate cancer	23/147	106/9375	2.01412 E-20	4.90102 E-19	1.85511 E-19	23	5293/5291/3551/1017/3480/572/5170/1956/2064/4314/2475/5290/207/2078/5594/5595/3320/208/10000/673/1147/4193/5604
**hsa05208**	Chemical carcinogenesis - reactive oxygen species	30/147	227/9375	6.81674 E-20	1.53114 E-18	5.79561 E-19	30	4233/5293/5291/5602/6714/3551/1432/25/5879/572/5580/5170/1956/5290/207/5747/3725/5594/5595/208/10000/5781/673/1147/3162/1544/5599/5604/2052/1728
**hsa05220**	Chronic myeloid leukemia	20/147	77/9375	1.48949 E-19	3.10665 E-18	1.17591 E-18	20	5293/5291/3551/25/1019/572/5290/7046/207/5594/5595/208/10000/5781/673/1147/3066/3065/4193/5604
**hsa04014**	Ras signaling pathway	30/147	238/9375	2.70932 E-19	4.88925 E-18	1.85065 E-18	30	998/4233/5293/5291/5602/5566/3815/3551/25/1436/5879/3480/2322/572/5578/3643/1956/3791/2321/5290/207/5594/5595/208/10000/5781/1147/5320/5599/5604

**hsa05221**	Acute myeloid leukemia	19/147	68/9375	2.74203 E-19	4.88925 E-18	1.85065 E-18	19	6198/5293/5291/3815/3551/1436/2322/572/2475/5290/207/5594/5595/208/10000/673/1147/4353/5604
**hsa04068**	FoxO signaling pathway	24/147	133/9375	2.84648 E-19	4.88925 E-18	1.85065 E-18	24	5293/5291/5602/3551/1432/1017/3480/5170/3643/1956/5290/7046/207/5594/5595/208/10000/673/1147/4193/23411/1901/5599/5604
**hsa04722**	Neurotrophin signaling pathway	23/147	120/9375	4.02568 E-19	6.46606 E-18	2.4475 E-18	23	998/5293/5291/5602/3551/1432/25/5879/572/5580/5170/5290/207/3725/5594/5595/208/10000/5781/673/51135/5599/5604
**hsa04510**	Focal adhesion	28/147	203/9375	4.20737 E-19	6.46606 E-18	2.4475 E-18	28	998/4233/5293/5291/5602/6093/6714/5879/3480/572/5578/5170/1956/2064/3791/2321/5290/207/5747/3725/5594/5595/208/10000/673/2534/5599/5604
**hsa04660**	T cell receptor signaling pathway	23/147	122/9375	5.96937 E-19	8.71528 E-18	3.29886 E-18	23	998/5293/5291/5602/3932/3551/1432/1019/5170/5290/207/3725/5594/5595/208/10000/5781/5788/1147/2534/10892/5599/5604
**hsa05223**	Non-small cell lung cancer	19/147	73/9375	1.21959 E-18	1.69581 E-17	6.41888 E-18	19	4233/5293/5291/1019/572/5578/5170/238/1956/2064/5290/207/5594/5595/208/10000/673/3718/5604
**hsa04062**	Chemokine signaling pathway	27/147	193/9375	1.33329 E-18	1.76964 E-17	6.69834 E-18	27	998/5293/5291/5294/6093/5566/3717/6714/3551/5879/572/5580/4067/5290/207/5747/5594/729230/5595/208/10000/673/1147/3718/5604/1234/2833
**hsa04380**	Osteoclast differentiation	24/147	142/9375	1.41665 E-18	1.79853 E-17	6.80771 E-18	24	5293/5291/5602/3932/3551/1432/1436/5879/695/3716/5290/7046/207/3725/5594/5595/208/100

								00/1147/2534/1513/5599/5604/7297
**hsa05167**	Kaposi sarcoma-associated herpesvirus infection	27/147	196/9375	2.00742E-18	2.44236E-17	9.2447E-18	27	3383/5293/5291/5294/5602/3717/6714/3551/1432/5743/1019/5879/4067/3716/2475/5290/207/3725/5594/5595/208/10000/1147/5599/5604/7297/1234
**hsa04926**	Relaxin signaling pathway	23/147	130/9375	2.68298E-18	3.13372E-17	1.18616E-17	23	5293/5291/5602/5566/6714/1432/5578/1956/4312/4842/4843/4846/5290/7046/207/3725/5594/5595/208/10000/4322/5599/5604
**hsa04664**	Fc epsilon RI signaling pathway	18/147	69/9375	9.92829E-18	1.11502E-16	4.22053E-17	18	5293/5291/5602/1432/5879/5578/4067/695/5170/5290/207/5594/5595/208/10000/2534/5599/5604
**hsa05135**	Yersinia infection	23/147	138/9375	1.08543E-17	1.17388E-16	4.44329E-17	23	998/5293/5291/5602/6093/3932/6714/3551/1432/5879/5290/207/5747/3725/5594/5595/3984/208/10000/1147/51135/5599/5604
**hsa04620**	Toll-like receptor signaling pathway	21/147	109/9375	1.40353E-17	1.46368E-16	5.54025E-17	21	5293/5291/5602/3551/1432/5879/3716/5290/207/3725/5594/5595/208/10000/1147/1513/51135/5599/5604/54106/7297
**hsa05418**	Fluid shear stress and atherosclerosis	23/147	142/9375	2.10746E-17	2.12199E-16	8.03205E-17	23	3383/6401/5293/5291/5602/6714/3551/1432/5879/3791/4846/5290/207/5747/3725/3320/208/10000/1147/3162/5599/1514/1728
**hsa05210**	Colorectal cancer	19/147	87/9375	4.45355E-17	4.33479E-16	1.64078E-16	19	6198/5293/5291/5602/5879/572/1956/2475/5290/7046/207/3725/5594/5595/208/10000/673/5599/5604
**hsa05166**	Human T-cell leukemia	27/147	224/9375	6.61973E-17	6.23536E-16	2.36018E-16	27	3383/5293/5291/11200/5602/5566/3932/3551/545/1017/1

	virus 1 infection							019/3716/7015/5290/7046/207/3725/5594/5595/208/10000/1147/3718/5599/5604/4316/706
**hsa05163**	Human cytomegalovirus infection	27/147	227/9375	9.33468E-17	8.5179E-16	3.22415E-16	27	6198/5293/5291/6093/5566/6714/3551/1432/5743/1019/5879/5578/1956/3716/2475/5290/207/5747/5594/5595/5732/208/10000/1147/4193/5604/1234
**hsa04625**	C-type lectin receptor signaling pathway	20/147	105/9375	1.10663E-16	9.79198E-16	3.70641E-16	20	5293/5291/5602/6714/3551/1432/5743/5580/5290/207/3725/5594/5595/208/10000/5781/1147/4193/10892/5599
**hsa05162**	Measles	22/147	136/9375	1.14102E-16	9.79931E-16	3.70918E-16	22	5293/5291/5602/3551/1017/1019/572/3716/1457/1459/5290/207/3725/208/10000/1147/3718/51135/5599/3312/54106/7297
**hsa04010**	MAPK signaling pathway	30/147	300/9375	1.95474E-16	1.63081E-15	6.17285E-16	30	998/4233/5602/5566/3815/3551/1432/1436/5879/3480/2322/5578/3643/1956/2064/3791/2321/7046/207/3725/5594/5595/208/10000/673/1147/51135/5599/3312/5604
**hsa05170**	Human immunodeficiency virus 1 infection	26/147	214/9375	2.18011E-16	1.76831E-15	6.69331E-16	26	6198/5293/5291/5602/3551/1432/545/5879/572/5578/983/2475/5290/207/5747/3725/5594/5595/3984/208/10000/1147/51135/5599/5604/1234
**hsa04066**	HIF-1 signaling pathway	20/147	110/9375	2.88647E-16	2.27797E-15	8.62247E-16	20	6198/5293/5291/3480/5578/3643/1956/2064/2475/4843/4846/2321/5290/207/5594/5595/208/10000/3162/5604
**hsa05230**	Central carbon metabolism in cancer	17/147	71/9375	4.11638E-16	3.12623E-15	1.18333E-15	17	4233/5293/5291/3815/2322/1956/2064/2475/5290/207/5594/5595/208/10000/2645/5604/3417

**hsa05145**	Toxoplasmosis	20/147	112/9375	4.17545E-16	3.12623E-15	1.18333E-15	20	5294/5602/3717/3551/1432/572/5170/3716/4843/207/5594/5595/208/10000/1147/51135/5599/3312/7297/1234
**hsa05169**	Epstein-Barr virus infection	25/147	205/9375	8.01165E-16	5.84851E-15	2.21375E-15	25	3383/5293/5291/5602/3551/1432/1017/1019/5879/4067/695/3716/5290/207/3725/208/10000/1147/3066/3065/4193/3718/51135/5599/7297
**hsa05225**	Hepatocellular carcinoma	23/147	170/9375	1.27536E-15	9.08305E-15	3.43807E-15	23	6198/4233/5293/5291/1019/3480/572/5578/1956/2475/7015/5290/7046/207/5594/5595/208/10000/673/1452/3162/5604/1728
**hsa04210**	Apoptosis	21/147	137/9375	1.84898E-15	1.28548E-14	4.86574E-15	21	5551/5293/5291/5602/3551/572/5170/5290/207/142/3725/5594/5595/208/10000/1147/1513/5599/2081/5604/1514
**hsa04071**	Sphingolipid signaling pathway	20/147	125/9375	3.86204E-15	2.62259E-14	9.9269E-15	20	5293/5291/5602/6093/1432/5879/5578/5170/4846/5290/207/4363/5594/5595/208/10000/1901/2534/5599/5604
**hsa04914**	Progesterone-mediated oocyte maturation	19/147	110/9375	4.54894E-15	3.01884E-14	1.14268E-14	19	6790/5293/5291/5602/5566/1432/1017/3480/983/5290/207/5594/5595/3320/208/10000/673/5599/5604
**hsa05211**	Renal cell carcinoma	16/147	70/9375	7.02153E-15	4.55619E-14	1.72459E-14	16	998/4233/5293/5291/5879/572/5290/207/3725/5594/5595/208/10000/5781/673/5604
**hsa04917**	Prolactin signaling pathway	16/147	71/9375	8.94441E-15	5.67775E-14	2.14911E-14	16	5293/5291/5602/3717/6714/1432/5290/207/5594/2099/5595/208/10000/2645/5599/5604
**hsa05231**	Choline metabolism in cancer	18/147	99/9375	9.74993E-15	6.0574E-14	2.29282E-14	18	6198/5293/5291/5602/5879/5578/5170/1956/2475/5290/207/3725/5594/5595

								/208/10000/5599/5604
**hsa05226**	Gastric cancer	21/147	150/9375	1.213E-14	7.37908E-14	2.79309E-14	21	6198/4233/5293/5291/1017/1956/2064/2475/7015/5290/7046/207/5243/5594/5595/208/10000/673/1452/6469/5604
**hsa05218**	Melanoma	16/147	73/9375	1.43414E-14	8.5463E-14	3.2349E-14	16	4233/5293/5291/1019/3480/572/1956/5290/207/5594/5595/208/10000/673/4193/5604
**hsa04015**	Rap1 signaling pathway	24/147	212/9375	1.75665E-14	1.02588E-13	3.88313E-14	24	998/4233/5293/5291/3815/6714/1432/135/1436/5879/3480/5578/3643/1956/3791/2321/5290/207/5594/5595/208/10000/673/5604
**hsa05142**	Chagas disease	18/147	103/9375	2.01935E-14	1.13194E-13	4.28458E-14	18	5293/5291/5602/3551/1432/4843/5290/7046/207/3725/5594/5595/208/10000/1147/51135/5599/54106
**hsa04213**	Longevity regulating pathway - multiple species	15/147	62/9375	2.04588E-14	1.13194E-13	4.28458E-14	15	6198/5293/5291/5566/3480/3643/2475/5290/207/208/10000/3066/3065/23411/3312
**hsa04668**	TNF signaling pathway	19/147	119/9375	2.05456E-14	1.13194E-13	4.28458E-14	19	3383/6401/5293/5291/5602/3551/1432/5743/4314/5290/207/3725/5594/5595/208/10000/1147/5599/5604
**hsa05165**	Human papillomavirus infection	29/147	333/9375	2.59372E-14	1.40253E-13	5.30879E-14	29	998/6198/5293/5291/5566/3551/545/5743/1017/1019/572/1956/3716/2475/7015/5290/207/5747/5594/5595/208/10000/1452/1147/3066/3065/4193/5604/7297
**hsa04910**	Insulin signaling pathway	20/147	138/9375	2.76234E-14	1.46655E-13	5.55111E-14	20	6198/5293/5291/5602/5566/3551/572/5170/3643/2475/5290/207/5594/5595/208/10000/673/2645/5599/5604
**hsa05214**	Glioma	16/147	76/9375	2.83097E-14	1.47615E-13	5.58745E-14	16	5293/5291/1019/3480/5578/1956/2475

								/5290/207/5594/5595/208/10000/673/4193/5604
**hsa04919**	Thyroid hormone signaling pathway	19/147	122/9375	3.29705E-14	1.68901E-13	6.39317E-14	19	5293/5291/5566/6714/572/5578/5170/2475/5290/207/5594/2099/5595/208/10000/3066/3065/4193/5604
**hsa05203**	Viral carcinogenesis	23/147	205/9375	7.92293E-14	3.98878E-13	1.50981E-13	23	998/5293/5291/5566/6714/1017/1019/5879/572/983/4067/3716/5290/3725/5594/5595/3066/3065/4193/3718/10013/55869/1234
**hsa05224**	Breast cancer	20/147	148/9375	1.08749E-13	5.38215E-13	2.03722E-13	20	6198/5293/5291/3815/1019/3480/1956/2064/2475/5290/207/3725/5594/2099/5595/208/10000/673/1452/5604
**hsa05131**	Shigellosis	25/147	253/9375	1.12269E-13	5.46376E-13	2.06812E-13	25	998/6198/5293/5291/5602/6093/6714/3551/1432/5879/5580/1956/2475/5290/207/5747/3725/5594/5595/208/10000/1147/4193/10892/5599
**hsa04666**	Fc gamma R-mediated phagocytosis	17/147	98/9375	1.27015E-13	6.08007E-13	2.3014E-13	17	998/6198/5293/5291/5879/5580/5578/4067/5290/207/5594/5595/3984/208/10000/5788/5604
**hsa05213**	Endometrial cancer	14/147	59/9375	2.10996E-13	9.93722E-13	3.76138E-13	14	5293/5291/572/5170/1956/2064/5290/207/5594/5595/208/10000/673/5604
**hsa05207**	Chemical carcinogenesis - receptor activation	23/147	217/9375	2.70034E-13	1.25159E-12	4.73744E-13	23	6198/5293/5291/5566/3717/6714/572/5578/1956/2475/5290/207/154/3725/5594/2099/5595/3320/208/10000/1544/5604/2052
**hsa04662**	B cell receptor signaling pathway	16/147	91/9375	5.57644E-13	2.54425E-12	9.63036E-13	16	5293/5291/3551/5879/4067/695/5290/207/3725/5594/5595/208/10000/1147/10892/5604
**hsa04611**	Platelet activation	18/147	126/9375	7.58906E-13	3.40924E-12	1.29045E-12	18	5293/5291/5294/6093/5566/6714/1432

								/4067/695/4846/5290/207/5594/5595/208/10000/2534/2147
**hsa04140**	Autophagy - animal	20/147	169/9375	1.39528E-12	6.17307E-12	2.3366E-12	20	6198/5293/5291/5602/5566/3480/572/5580/5170/2475/5290/207/5594/5595/208/10000/5599/2081/5604/1514
**hsa05160**	Hepatitis C	19/147	156/9375	3.14724E-12	1.37163E-11	5.19184E-12	19	5293/5291/3551/1017/1019/572/1956/3716/5290/207/5594/949/5595/208/10000/673/1147/5604/7297
**hsa04218**	Cellular senescence	19/147	157/9375	3.5336E-12	1.51737E-11	5.74347E-12	19	5293/5291/11200/1432/545/1017/1019/983/2475/5290/7046/207/5594/5595/208/10000/4193/23411/5604
**hsa04915**	Estrogen signaling pathway	18/147	139/9375	4.23829E-12	1.79359E-11	6.78902E-12	18	5293/5291/5566/6714/5580/1956/4846/5290/207/3725/5594/2099/5595/3320/208/10000/3312/5604
**hsa04930**	Type II diabetes mellitus	12/147	47/9375	4.58462E-12	1.91244E-11	7.23887E-12	12	5293/5291/5602/3551/5580/3643/2475/5290/5594/5595/2645/5599
**hsa04517**	IgSF CAM signaling	25/147	299/9375	4.90703E-12	2.0181E-11	7.63882E-12	25	998/3383/5293/5291/5602/3932/6714/1432/25/5879/5578/4067/5170/3791/5290/207/5747/5594/5595/208/10000/5781/2534/5599/5604
**hsa04935**	Growth hormone synthesis, secretion and action	17/147	122/9375	5.18637E-12	2.10336E-11	7.96153E-12	17	5293/5291/5602/5566/3717/1432/5578/2475/5290/207/5747/5594/5595/208/10000/5599/5604
**hsa04659**	Th17 cell differentiation	16/147	109/9375	1.00497E-11	3.96554E-11	1.50102E-11	16	5602/3717/3932/3551/1432/3716/2475/7046/3725/5594/5595/3320/1147/3718/5599/7297
**hsa04931**	Insulin resistance	16/147	109/9375	1.00497E-11	3.96554E-11	1.50102E-11	16	6198/5293/5291/5602/3551/5580/5170/3643/2475/4846/5

								290/207/208/10000/5781/5599
**hsa04520**	Adherens junction	15/147	93/9375	1.13553E-11	4.421E-11	1.67341E-11	15	998/4233/6093/6714/5879/3480/3643/1956/2064/1457/1459/7046/5594/5595/2534
**hsa04072**	Phospholipase D signaling pathway	18/147	149/9375	1.40503E-11	5.39826E-11	2.04332E-11	18	5293/5291/5294/3815/5578/3643/1956/2475/5290/207/5594/5595/208/10000/5781/2534/5604/2147
**hsa04613**	Neutrophil extracellular trap formation	20/147	195/9375	2.03801E-11	7.72855E-11	2.92537E-11	20	5293/5291/6714/1432/5879/5578/2475/1991/5290/207/5594/5595/208/10000/3066/3065/4353/5604/10013/55869
**hsa04518**	Integrin signaling	18/147	154/9375	2.46958E-11	9.24509E-11	3.4994E-11	18	998/4233/3383/5293/5291/6714/5879/3480/1956/2064/2475/3791/2321/5290/207/5747/208/10000
**hsa05132**	Salmonella infection	22/147	248/9375	3.36223E-11	1.24275E-10	4.70398E-11	22	998/5293/5291/5294/5602/3551/1432/5879/5290/207/3725/5594/5595/3320/208/10000/5788/1147/51135/5599/5604/54106
**hsa04150**	mTOR signaling pathway	18/147	158/9375	3.81822E-11	1.39365E-10	5.27518E-11	18	6198/5293/5291/3551/3480/5578/5170/3643/2475/5290/207/5594/5595/208/10000/673/1147/5604
**hsa05120**	Epithelial cell signaling in Helicobacter pylori infection	13/147	71/9375	5.40455E-11	1.94831E-10	7.37463E-11	13	998/4233/5602/6714/3551/1432/5879/4067/1956/3725/5781/1147/5599
**hsa05164**	Influenza A	18/147	173/9375	1.75271E-10	6.24136E-10	2.36245E-10	18	3383/5293/5291/3717/3551/1019/5578/3716/5290/207/5594/5595/208/10000/1147/51135/5604/7297
**hsa05010**	Alzheimer disease	26/147	388/9375	2.52846E-10	8.8953E-10	3.367E-10	26	5293/5291/5602/7494/3551/5743/572/3643/1457/1459/2475/4842/4843/5290

								/207/5594/5595/208/10000/673/1452/1147/5599/2081/5604/4311
**hsa04725**	Cholinergic synapse	15/147	115/9375	2.59678E-10	9.02691E-10	3.41682E-10	15	5293/5291/5294/5566/3717/43/5578/5290/207/5594/5595/208/10000/2534/5604
**hsa04024**	cAMP signaling pathway	20/147	226/9375	2.98602E-10	1.02579E-09	3.88275E-10	20	5293/5291/5602/6093/5566/135/5879/572/5290/207/154/3725/5594/5595/5732/208/10000/673/5599/5604
**hsa05415**	Diabetic cardiomyopathy	19/147	205/9375	3.91683E-10	1.3299E-09	5.03387E-10	19	5293/5291/5602/1432/5879/5580/5578/3643/2475/4846/5290/7046/5972/207/142/208/10000/2936/5599
**hsa04360**	Axon guidance	18/147	184/9375	4.86145E-10	1.62174E-09	6.13853E-10	18	998/4233/5293/5291/6093/6714/25/5879/5578/5170/5290/5747/5594/5595/3984/5781/2534/6469
**hsa05219**	Bladder cancer	10/147	41/9375	4.88744E-10	1.62174E-09	6.13853E-10	10	6714/1019/1956/2064/4312/5594/5595/673/4193/5604
**hsa05171**	Coronavirus disease - COVID-19	20/147	238/9375	7.50181E-10	2.46127E-09	9.31626E-10	20	5293/5291/5602/3551/1432/5578/1956/3716/4314/4312/5290/3725/5594/5595/1675/1147/51135/5599/7297/2147
**hsa04211**	Longevity regulating pathway	13/147	90/9375	1.18492E-09	3.8444E-09	1.45516E-09	13	6198/5293/5291/5566/3480/3643/10919/2475/5290/207/208/10000/23411
**hsa01524**	Platinum drug resistance	12/147	75/9375	1.57469E-09	5.05284E-09	1.91257E-09	12	5293/5291/572/5170/2064/5290/207/5594/5595/208/10000/4193
**hsa04658**	Th1 and Th2 cell differentiation	13/147	93/9375	1.79772E-09	5.5844E-09	2.11378E-09	13	5602/3717/3932/3551/1432/3716/3725/5594/5595/1147/3718/5599/7297
**hsa04912**	GnRH signaling pathway	13/147	93/9375	1.79772E-09	5.5844E-09	2.11378E-09	13	998/5602/5566/6714/1432/5580/5578/1956/3725/5594/5595/5599/5604
**hsa05222**	Small cell lung cancer	13/147	93/9375	1.79772E-09	5.5844E-09	2.11378E-09	13	5293/5291/3551/5743/1017/1019/4843

								/5290/207/5747/208/10000/1147
**hsa04657**	IL-17 signaling pathway	13/147	94/9375	2.05846E-09	6.32704E-09	2.39488E-09	13	5602/3551/1432/5743/4314/4312/3725/5594/5595/3320/1147/4322/5599
**hsa04932**	Non-alcoholic fatty liver disease	16/147	157/9375	2.62274E-09	7.9203E-09	2.99795E-09	16	998/5293/5291/5602/7494/3551/1432/5879/3643/5290/207/3725/208/10000/5599/2081
**hsa05168**	Herpes simplex virus 1 infection	17/147	180/9375	2.63106E-09	7.9203E-09	2.99795E-09	17	5293/5291/3717/6714/3551/572/3716/2475/5290/207/208/10000/5781/1147/51135/54106/7297
**hsa05206**	MicroRNAs in cancer	22/147	323/9375	5.28214E-09	1.57386E-08	5.9573E-09	22	4233/5293/5291/6093/3551/25/5743/5578/1956/2064/2475/5290/5243/4363/5594/5595/3066/3065/4193/3162/23411/5604
**hsa05130**	Pathogenic Escherichia coli infection	17/147	200/9375	1.30937E-08	3.86197E-08	1.46181E-08	17	998/5602/6093/6714/3551/1432/25/5879/3725/5594/5595/5781/1147/2534/51135/5599/2147
**hsa04650**	Natural killer cell mediated cytotoxicity	14/147	133/9375	1.80292E-08	5.26453E-08	1.9927E-08	14	5551/3383/5293/5291/3932/5879/5578/5290/5594/5595/5781/673/2534/5604
**hsa04810**	Regulation of actin cytoskeleton	18/147	232/9375	1.99942E-08	5.78051E-08	2.18801E-08	18	998/5293/5291/6093/6714/5879/1956/5290/207/5747/5594/5595/3984/208/10000/673/5604/2147
**hsa05152**	Tuberculosis	16/147	181/9375	2.06483E-08	5.91107E-08	2.23743E-08	16	5602/3717/6714/1432/572/3716/4843/207/5594/5595/208/10000/10892/51135/5599/54106
**hsa04923**	Regulation of lipolysis in adipocytes	10/147	59/9375	2.15285E-08	6.10324E-08	2.31017E-08	10	5293/5291/5566/5743/3643/5290/207/154/208/10000
**hsa04371**	Apelin signaling pathway	14/147	140/9375	3.49152E-08	9.80312E-08	3.71062E-08	14	6198/5294/5566/2475/4842/4843/4846/7046/207/5594/5595/208/10000/5604
**hsa04750**	Inflammatory mediator	12/147	99/9375	4.03846E-08	1.12308E-07	4.25101E-08	12	5293/5291/5602/5566/6714/1432/5580

	regulation of TRP channels							/5578/5290/5732/7442/5599
**hsa04550**	Signaling pathways regulating pluripotency of stem cells	14/147	144/9375	5.00479E-08	1.37868E-07	5.2185E-08	14	5293/5291/3717/1432/3480/3716/5290/207/5594/5595/208/10000/3718/5604
**hsa04929**	GnRH secretion	10/147	65/9375	5.66105E-08	1.54489E-07	5.84762E-08	10	5293/5291/5578/5290/207/5594/5595/208/10000/5604
**hsa04064**	NF-kappa B signaling pathway	12/147	105/9375	7.8672E-08	2.12706E-07	8.05123E-08	12	3383/3932/3551/5743/4067/695/1457/1459/142/1147/10892/51135
**hsa04960**	Aldosterone-regulated sodium reabsorption	8/147	38/9375	9.9181E-08	2.65696E-07	1.0057E-07	8	5293/5291/5578/5170/3643/5290/5594/5595
**hsa04920**	Adipocytokine signaling pathway	10/147	70/9375	1.17339E-07	3.11482E-07	1.179E-07	10	5602/3717/3551/2475/207/208/10000/5781/1147/5599
**hsa04726**	Serotonergic synapse	12/147	115/9375	2.17376E-07	5.71835E-07	2.16448E-07	12	5566/5743/1565/5578/6532/4128/5594/5595/673/5604/1559/1557
**hsa05020**	Prion disease	18/147	275/9375	2.68036E-07	6.98808E-07	2.64509E-07	18	5293/5291/5602/5566/1432/5879/572/5580/1457/1459/5290/5594/5595/2534/5599/3312/3309/5621
**hsa04152**	AMPK signaling pathway	12/147	122/9375	4.16562E-07	1.07643E-06	4.07443E-07	12	6198/5293/5291/3480/5170/3643/2475/5290/207/208/10000/23411
**hsa04921**	Oxytocin signaling pathway	13/147	154/9375	7.98817E-07	2.04609E-06	7.74476E-07	13	5294/6093/5566/6714/5743/5578/1956/4846/3725/5594/5595/952/5604
**hsa04148**	Efferocytosis	13/147	157/9375	9.96169E-07	2.5294E-06	9.57417E-07	13	3717/1432/5743/5879/7301/5747/5594/5595/5732/5781/23411/1901/5604
**hsa05022**	Pathways of neurodegeneration - multiple diseases	23/147	480/9375	1.43348E-06	3.60841E-06	1.36584E-06	23	5602/7494/1432/120892/5743/5879/6531/572/5578/1457/1459/2475/4842/4843/5594/5595/673/1452/5599/2081/3309/5604/5621
**hsa04670**	Leukocyte transendothelial migration	11/147	116/9375	1.85879E-06	4.63903E-06	1.75594E-06	11	998/3383/5293/5291/6093/1432/5879/5578/5290/5747/5781

**hsa04630**	JAK-STAT signaling pathway	13/147	168/9375	2.1451 E-06	5.3082E -06	2.00923 E-06	13	5293/5291/3717/1956/3716/2475/5290/207/208/10000/5781/3718/7297
**hsa05017**	Spinocerebellar ataxia	12/147	144/9375	2.47566 E-06	6.07472 E-06	2.29937 E-06	12	5293/5291/5602/7494/5578/2475/5290/207/208/10000/5599/2081
**hsa05202**	Transcriptional misregulation in cancer	14/147	201/9375	3.00094 E-06	7.30228 E-06	2.76402 E-06	14	4233/1025/1436/3480/2322/4314/2321/1991/5747/2078/3066/3065/4193/4353
**hsa05140**	Leishmaniasis	9/147	78/9375	3.25729 E-06	7.86056 E-06	2.97534 E-06	9	3717/1432/5743/3716/4843/3725/5594/5595/51135
**hsa04730**	Long-term depression	8/147	60/9375	3.8783 E-06	9.2825E -06	3.51356 E-06	8	3480/5578/4067/4842/5594/5595/673/5604
**hsa04621**	NOD-like receptor signaling pathway	13/147	187/9375	7.03282 E-06	1.66958 E-05	6.31961 E-06	13	5602/3551/1432/5580/3716/3725/5594/5595/3320/1147/51135/5599/7297
**hsa04973**	Carbohydrate digestion and absorption	7/147	48/9375	8.68307 E-06	2.04472 E-05	7.73958 E-06	7	5293/5291/5290/207/208/10000/6523
**hsa04022**	cGMP-PKG signaling pathway	12/147	166/9375	1.08213 E-05	2.52785 E-05	9.5683 E-06	12	5294/6093/572/3643/4846/207/154/5594/5595/208/10000/5604
**hsa05031**	Amphetamine addiction	8/147	69/9375	1.12528 E-05	2.6078E -05	9.8709 E-06	8	5566/6531/5578/3725/4128/3066/3065/23411
**hsa04936**	Alcoholic liver disease	11/147	144/9375	1.51962 E-05	3.49393 E-05	1.32251 E-05	11	5602/3551/1432/207/208/10000/1147/23411/217/51135/5599
**hsa05100**	Bacterial invasion of epithelial cells	8/147	78/9375	2.80192 E-05	6.34233 E-05	2.40067 E-05	8	998/4233/5293/5291/6714/5879/5290/5747
**hsa05133**	Pertussis	8/147	78/9375	2.80192 E-05	6.34233 E-05	2.40067 E-05	8	5602/1432/4843/3725/5594/5595/51135/5599
**hsa04110**	Cell cycle	11/147	158/9375	3.62273 E-05	8.1372E -05	3.08005 E-05	11	11200/545/25/1017/1019/983/5111/3066/3065/4193/55869
**hsa04728**	Dopaminergic synapse	10/147	133/9375	4.30298 E-05	9.59138 E-05	3.63048 E-05	10	5602/5566/1432/6531/5578/207/4128/208/10000/5599
**hsa05034**	Alcoholism	12/147	191/9375	4.39994 E-05	9.73321 E-05	3.68416 E-05	12	5566/135/6531/4128/5594/5595/673/3

								066/3065/5604/10013/55869
**hsa04270**	Vascular smooth muscle contraction	10/147	134/9375	4.5884E-05	0.000100738	3.81307E-05	10	6093/5566/135/5580/5578/5594/5595/673/5320/5604
**hsa04519**	Cadherin signaling	18/147	404/9375	5.82798E-05	0.000126998	4.80705E-05	18	998/4233/5602/6093/3551/1432/5879/3480/1956/2064/3791/5747/5594/5595/1452/1147/5599/5604
**hsa04530**	Tight junction	11/147	170/9375	7.08253E-05	0.000153193	5.79856E-05	11	998/5602/6093/5566/6714/1019/5879/2064/3725/5111/5599
**hsa04540**	Gap junction	8/147	89/9375	7.29993E-05	0.000156734	5.93261E-05	8	5566/6714/5578/983/1956/5594/5595/5604
**hsa01523**	Antifolate resistance	5/147	30/9375	9.19724E-05	0.000196029	7.41998E-05	5	7298/1719/3551/4363/1147
**hsa04310**	Wnt signaling pathway	11/147	178/9375	0.000107151	0.000226725	8.58189E-05	11	5602/5566/5879/5578/1457/1459/3725/1452/23411/5599/4316
**hsa04261**	Adrenergic signaling in cardiomyocytes	10/147	154/9375	0.000147869	0.000310631	0.000117578	10	5294/5566/1432/5578/207/154/5594/5595/208/10000
**hsa04020**	Calcium signaling pathway	13/147	254/9375	0.000171885	0.000358504	0.000135699	13	4233/5566/135/5578/1956/2064/3791/4842/4843/4846/2321/154/952
**hsa04114**	Oocyte meiosis	9/147	138/9375	0.000308655	0.000639201	0.000241947	9	6790/5566/1432/1017/3480/983/5594/5595/5604
**hsa04928**	Parathyroid hormone synthesis, secretion and action	8/147	115/9375	0.000434189	0.000892839	0.000337953	8	5566/5578/1956/5594/5595/673/4322/5604
**hsa04720**	Long-term potentiation	6/147	67/9375	0.000609632	0.001244843	0.000471191	6	5566/5578/5594/5595/673/5604
**hsa04081**	Hormone signaling	11/147	219/9375	0.000643389	0.00130465	0.000493829	11	5293/5291/5566/3717/6714/3480/3643/4842/5290/154/2099
**hsa05323**	Rheumatoid arthritis	7/147	95/9375	0.000705766	0.001421266	0.00053797	7	3383/4314/4312/2321/3725/1513/1514
**hsa04934**	Cushing syndrome	9/147	155/9375	0.000722417	0.001444834	0.000546891	9	5566/1017/1019/1956/5594/949/5595/673/5604
**hsa05416**	Viral myocarditis	6/147	70/9375	0.000770745	0.001531004	0.000579508	6	5551/3383/25/5879/4842/2534

**hsa00330**	Arginine and proline metabolism	5/147	50/9375	0.001063259	0.002097781	0.000794041	5	4842/4843/4846/4128/217
**hsa04115**	p53 signaling pathway	6/147	75/9375	0.001110246	0.002175785	0.000823567	6	11200/545/1017/1019/983/4193
**hsa05146**	Amoebiasis	7/147	103/9375	0.001140589	0.002220347	0.000840434	7	5293/5291/5566/5578/4843/5290/5747
**hsa00591**	Linoleic acid metabolism	4/147	30/9375	0.001157961	0.002239235	0.000847584	4	1544/5320/1559/1557
**hsa04913**	Ovarian steroidogenesis	5/147	52/9375	0.001271947	0.002443476	0.000924892	5	5566/5743/3480/3643/949
**hsa04350**	TGF-beta signaling pathway	7/147	110/9375	0.001672861	0.00319265	0.001208465	7	6198/6093/7046/5594/5595/3066/3065
**hsa04723**	Retrograde endocannabinoid signaling	8/147	149/9375	0.002339225	0.004435414	0.00167887	8	5602/5566/1432/5743/5578/5594/5595/5599
**hsa05143**	African trypanosomiasis	4/147	37/9375	0.00256361	0.004798551	0.001816323	4	3383/6401/5578/54106
**hsa05216**	Thyroid cancer	4/147	37/9375	0.00256361	0.004798551	0.001816323	4	5594/5595/673/5604
**hsa05340**	Primary immunodeficiency	4/147	38/9375	0.002830754	0.005264843	0.001992821	4	3932/695/5788/3718
**hsa05204**	Chemical carcinogenesis - DNA adducts	5/147	70/9375	0.004730483	0.008742412	0.003309132	5	5743/1544/2052/1559/1557
**hsa04217**	Necroptosis	8/147	168/9375	0.004873089	0.00894932	0.00338745	8	5602/3717/3716/142/3320/3718/5599/7297
**hsa04916**	Melanogenesis	6/147	101/9375	0.005015281	0.009152887	0.003464503	6	5566/3815/5578/5594/5595/5604
**hsa00220**	Arginine biosynthesis	3/147	23/9375	0.005314794	0.009614634	0.003639281	3	4842/4843/4846
**hsa04622**	RIG-I-like receptor signaling pathway	5/147	72/9375	0.005334146	0.009614634	0.003639281	5	5602/3551/1432/1147/5599
**hsa00982**	Drug metabolism - cytochrome P450	5/147	73/9375	0.005655558	0.010131428	0.003834895	5	1565/4128/1544/1559/1557
**hsa04141**	Protein processing in endoplasmic reticulum	8/147	174/9375	0.006002399	0.010687198	0.004045262	8	5602/7494/3320/11253/5599/2081/3312/3309
**hsa04137**	Mitophagy - animal	6/147	105/9375	0.006053088	0.010712132	0.00405547	6	5602/6714/1457/1459/3725/5599

**hsa04918**	Thyroid hormone synthesis	5/147	75/9375	0.006338994	0.011150519	0.004220636	5	5566/5578/2936/7276/3309
**hsa04922**	Glucagon signaling pathway	6/147	107/9375	0.006627005	0.011587338	0.004385979	6	5566/207/208/10000/2645/23411
**hsa05030**	Cocaine addiction	4/147	49/9375	0.007115628	0.012367639	0.004681334	4	5566/6531/3725/4128
**hsa05144**	Malaria	4/147	50/9375	0.007641921	0.013203792	0.00499783	4	4233/3383/6401/54106
**hsa05012**	Parkinson disease	10/147	268/9375	0.009443687	0.016220922	0.006139859	10	5602/7494/5566/120892/135/6531/4128/5599/2081/3309
**hsa04981**	Folate transport and metabolism	3/147	31/9375	0.012314356	0.021028023	0.007959417	3	7298/1719/4363
**hsa04976**	Bile secretion	5/147	90/9375	0.0134028	0.02275359	0.00861257	5	5566/5243/949/6523/2052
**hsa04970**	Salivary secretion	5/147	93/9375	0.015272589	0.025778011	0.009757358	5	5566/5578/4842/154/952
**hsa00590**	Arachidonic acid metabolism	4/147	63/9375	0.016914576	0.02838538	0.010744286	4	5743/5320/1559/1557
**hsa04713**	Circadian entrainment	5/147	97/9375	0.018026663	0.030078774	0.011385261	5	5566/5578/4842/5594/5595
**hsa04640**	Hematopoietic cell lineage	5/147	100/9375	0.020295143	0.033671487	0.012745155	5	3815/1436/2322/952/4311
**hsa00760**	Nicotinate and nicotinamide metabolism	3/147	38/9375	0.021349191	0.035220134	0.013331341	3	22933/952/23411
**hsa04924**	Renin secretion	4/147	69/9375	0.0228539	0.037490668	0.014190772	4	5566/5972/154/5732
**hsa00380**	Tryptophan metabolism	3/147	42/9375	0.02777116	0.045302675	0.017147732	3	4128/1544/217

Table S8. Selection rationale for the 10 KEGG pathways visualized in the compound–target–pathway network and Sankey diagram.

**Pathway ID**	**Pathway Description**	**Adjusted p-value**	**Connected Core Target(s)**	**Relevance to EV-A71**
hsa04151	PI3K-Akt signaling pathway	4.18e-19	MAPK1, MAPK3, JUN	Viral replication, cell survival
hsa05161	Hepatitis B	1.40e-20	MAPK1, MAPK3, JUN	Viral infection, innate immunity
hsa04657	IL-17 signaling pathway	1.57e-14	MAPK1, MAPK3, JUN	Inflammatory response
hsa04668	TNF signaling pathway	2.06e-09	MAPK1, MAPK3, JUN	Inflammatory response, apoptosis
hsa04012	ErbB signaling pathway	2.89e-21	MAPK1, MAPK3	Cell proliferation, MAPK cascade
hsa01521	EGFR inhibitor resistance	4.99e-22	MAPK1, MAPK3	Growth factor signaling, MAPK pathway

hsa05417	Lipid and atherosclerosis	2.80e-26	MAPK1, MAPK3	Inflammation, immune response
hsa01522	Endocrine resistance	8.72e-27	MAPK1, MAPK3	Signaling crosstalk, MAPK pathway
hsa05205	Proteoglycans in cancer	2.09e-22	MAPK1, MAPK3	Cell signaling, inflammation
hsa05235	PD-L1 expression pathway	1.31e-23	MAPK1, MAPK3	Immune checkpoint, viral evasion

Note: Pathways were selected based on three criteria: (1) statistical significance (adjusted p-value < 0.05), (2) biological relevance to EV-A71 infection, and (3) connectivity to at least one of the five core targets (MAPK1, MAPK3, JUN, AURKB, MAPK8). Adjusted p-values were calculated using the Benjamini-Hochberg method.

Table S9. Risk of Bias Summary.

Author(year)	A	B	C	D	E	F	G	H	I	J	Total
**Weng et al.(2025)**	?	+	?	?	?	?	+	+	+	?	4
**Cui et al. (2022)**	?	+	?	?	?	+	?	+	+	+	5
**Yang et al. (2020)**	?	?	?	?	?	+	?	+	+	?	3
**Tang et al. (2020)**	?	+	?	?	?	?	?	+	+	+	4
**Wang et al. (2019)**	?	+	?	?	?	?	?	+	+	+	4
**Chen et al. (2018)**	?	?	?	?	?	?	?	+	+	+	3
**Yang et al. （2015）**	?	?	?	?	?	?	?	+	+	?	2
**Yang et al. (2012)**	?	?	?	?	?	?	?	+	?	+	2
**Liu et al. (2011)**	?	?	?	?	?	?	?	+	+	?	2

(A) Sequence generation. (B) Baseline characteristics. (C) Allocation concealment. (D) Random housing. (E) Blinding of experimentalists. (F) Random outcome assessment. (G) Blinding of outcome assessors. (H) Incomplete outcome data. (I) Selective outcome reporting. (J) Other sources of bias. +: indicates low risk; -: indicates high risk; ?: indicates unclear risk.

Table S10 Quality of evidence based on GRADE's tool.

Outcomes	**Anticipated absolute effects** (95% CI)		Relative effect (95% CI)	№ of participants (studies)	Certainty of the evidence (GRADE)
	**Risk with NS**	**Risk with Alkaloids**			
survival rate	33 per 1,000	**507 per 1,000** (260 to 751)	**OR 30.62** (10.44 to 89.82)	239 (8 RCTs)	⊕⊕⊕◯ Moderate[a]
body weight	The mean body weight was **0**	MD **2.68 higher** (2 higher to 3.37 higher)	-	79 (4 RCTs)	⊕◯◯◯ Very low[a,c,]
clinical score	The mean clinical score was **0**	SMD **2.86 lower** (3.55 lower to 2.17 lower)	-	155 (8 RCTs)	⊕⊕◯◯ Low[a,c,]
VPI expression	The mean VPI expression was **0**	MD **4.93 lower** (14.28 lower to 4.41 higher)	-	14 (2 RCTs)	⊕◯◯◯ Very low[a,b,c,d]

Outcomes	Anticipated absolute effects (95% CI)		Relative effect (95% CI)	№ of participants (studies)	Certainty of the evidence (GRADE)
	**Risk with NS**	**Risk with Alkaloids**			
brain viral load	The mean brain viral load was **0**	MD **2.38 lower** (4.16 lower to 0.6 lower)	-	26 (3 RCTs)	⊕◯◯◯ Very low[a,b,c]
hind limb viral load	The mean brain viral load was **0**	MD **3.67 lower** (6.12 lower to 1.22 lower)	-	104 (6 RCTs)	⊕⊕◯◯ Low[a,c,]

CI: Confidence interval; **MD:** Mean difference; **OR:** Odds ratio

GRADE Working Group grades of evidence

High certainty: we are very confident that the true effect lies close to that of the estimate of the effect.

Moderate certainty: we are moderately confident in the effect estimate: the true effect is likely to be close to the estimate of the effect, but there is a possibility that it is substantially different.

Low certainty: our confidence in the effect estimate is limited: the true effect may be substantially different from the estimate of the effect.

Very low certainty: we have very little confidence in the effect estimate: the true effect is likely to be substantially different from the estimate of effect.

Explanations a.high risk of bias from selecting the reported results and carryover effects; b.small sample size; c.high heterogeneity; d.95% CI fails to exclude important benefit or important harm

Table S11 Complete molecular docking results for all compound–target pairs assessed

Compound	MAPK1	MAPK3	JUN	AURKB	MAPK8
Berberine	−9.3	−9.1	-5.1	-5.5	-5.6
Cepharanthine	-5.2	-4.9	-5.7	-5.3	-4.8
Emetine	-5.7	-5.2	-5.0	−7.9	-4.2
Harmine	-4.9	-5.7	-5.3	-4.7	−6.7
Lycorine	−7.6	-5.7	-5.3	-5.7	-5.4
Matrine	-5.1	-5.2	−6.8	-4.7	-5.4
Oxysophocarpine	−5.8	-5.6	-4.6	-4.9	-5.6
